# The Evolving Clonal, Plasmid-Mediated Resistome, Virulome and Therapeutic Landscape of Carbapenem-Resistant *Klebsiella pneumoniae* in Oman

**DOI:** 10.3390/antibiotics15090844

**Published:** 2026-08-31

**Authors:** Arwa Al Rujaibi, Zaaima Al Jabri, Azza Mohammed Al Mamari, Amira ElBaradei, Hafidha Al-Hattali, Faiza Syed, Zakariya Al Muharrmi, Amina Al-Jardani, Meher Rizvi

**Affiliations:** 1 Fisheries Water Resources, Central Laboratory of Animal Health, Ministry of Agriculture, Saal, P.O. Box 467, Muscat 100, Oman; arwa.alrejebi@mafwr.gov.om; 2 Department of Microbiology and Immunology, College of Medicine and Health Sciences, Sultan Qaboos University, Muscat 123, Oman; zaeema@squ.edu.om (Z.A.J.); a.elbaradei1@squ.edu.om (A.E.); 3 Department of Biomedical Science, College of Medicine and Health Sciences, Sultan Qaboos University, Muscat 123, Oman; h.hattali@squ.edu.om; 4 DASH to Protect Antibiotics, Muscat 111, Oman; 5 Department of Microbiology and Immunology, Sultan Qaboos University Hospital and University Medical City, Muscat 112, Oman; 6 Central Public Health Laboratories, Center for Disease Control and Prevention, Ministry of Health, Muscat 123, Oman; amina.aljardani@moh.gov.om

**Keywords:** carbapenem-resistant *K. pneumoniae*, AMR, resistome, virulome, plasmidome, newer antimicrobials, antimicrobial synergistic combinations

## Abstract

Background: Carbapenem-resistant *Klebsiella pneumoniae* (CRKP) increasingly combines high-risk clonal expansion, mobile resistance platforms and limited treatment options. We investigated the genomic epidemiology, resistance and virulence architecture, plasmid backbones, and therapeutic vulnerabilities of CRKP circulating in Oman. Methods: Between 2021 and 2024, 135 non-duplicate CRKP isolates were recovered from diverse clinical specimens. New antimicrobial agents were evaluated phenotypically, while 38 representative extensively drug-resistant (XDR)/pan-drug resistant (PDR) isolates underwent whole-genome sequencing (WGS) for multilocus sequence typing (MLST), capsular typing, resistome, virulome, plasmid and mobile genetic elements (MGEs) analysis. In vitro synergy of ceftazidime–avibactam/aztreonam, meropenem/fosfomycin and amikacin/fosfomycin was assessed using gradient diffusion-based FICI. Results: WGS revealed a striking shift towards OXA-232-producing ST-2096, which dominated the sequenced collection and carried KL64 with a conserved multidrug-resistant backbone. NDM-5/ST147 and NDM-1 + KPC-2/ST11 formed distinct high-risk lineages with broader extended-spectrum β-lactamase (ESBL) repertoires, greater plasmid heterogeneity and, in co-producers, the highest MGE burden. Across isolates, resistance was reinforced by widespread blaCTX-M variants, *armA*, *aac(6′)-Ib-cr*, *fosA*, porin alterations and fluoroquinolone-resistance mutations, while core virulence and fitness loci including *fimH*, *mrkA*, *iutA*, *fyuA* and *irp2* were widely retained. Cefiderocol showed the most consistent in vitro activity across carbapenemase groups, and eravacycline remained active, whereas plazomicin and fosfomycin activity was compromised by methyltransferase and fos genes. Ceftazidime–avibactam/aztreonam demonstrated universal synergy, while meropenem/fosfomycin and amikacin/fosfomycin showed limited, carbapenemase-dependent activity. Conclusions: CRKP in Oman is characterised by convergent clonal expansion, plasmid-mediated resistance, retained virulence potential and narrowing therapeutic options. Integrated genomic surveillance with carbapenemase-directed susceptibility and synergy testing is essential to guide precision antimicrobial stewardship in high-risk healthcare settings and inform early infection prevention responses to emerging regional CRKP lineages.

## 1. Introduction

The rise in extensively drug-resistant (XDR) Gram-negative bacilli (GNB) has emerged as one of the most pressing challenges in modern medicine. Of particular concern are carbapenem-resistant *Klebsiella pneumoniae* (CRKP), which have been recognised by the World Health Organisation (WHO) as a critical priority pathogen due to high mortality rates and limited treatment options [1].

Based on amino acid sequence homology, β-lactamases are classified according to the Ambler system into four molecular classes: A, B, C, and D. Classes A, C, and D are serine β-lactamases, in which a serine residue at the active site is essential for hydrolysis of the β-lactam ring, whereas class B enzymes are metallo-β-lactamases (MBLs) that require zinc ions for catalytic activity [2]. Class A includes extended-spectrum β-lactamases (ESBLs), such as TEM, SHV, and CTX-M enzymes, as well as carbapenemases such as *Klebsiella pneumoniae* carbapenemase (KPC). Class B comprises MBLs, including New Delhi metallo-β-lactamase (NDM), Verona integron-encoded metallo-β-lactamase (VIM), and imipenemase (IMP). Class C consists predominantly of AmpC-type cephalosporinases, including chromosomal and plasmid-mediated enzymes such as CMY and DHA, whereas class D comprises oxacillinase (OXA)-type β-lactamases, including the clinically important OXA-48-like carbapenemases. Carbapenem resistance in Enterobacterales is most commonly mediated by the production of carbapenemases, including KPC, NDM, and OXA-48-like enzymes, although other mechanisms, such as porin alterations or loss and increased efflux activity, may also contribute to resistance [3]. These genes are frequently carried on mobile genetic elements (MGEs), facilitating rapid horizontal transmission within and between bacterial species [4].

Gulf Cooperation Council countries report the growing prevalence and genetic diversity of CRKP clones, alongside significant challenges in containment and treatment [5,6,7]. The dissemination of high-risk clones such as ST14, ST231, and ST147 has been documented across Saudi Arabia, Oman, the United Arab Emirates, Kuwait, and Bahrain [5,7]. Understanding the prevalent clades is essential not only for surveillance purposes but also for optimising empiric treatment and instituting appropriate infection prevention and control [8,9].

Ceftazidime–avibactam (CZA), imipenem–relebactam (IMR), meropenem–vaborbactam (MPV), cefiderocol (CFD), eravacycline (ERV) and plazomicin (PLZ) have recently been introduced for management of extremely drug-resistant (XDR) GNB [10,11]. CFD possesses structural similarities with cefepime and ceftazidime but incorporates a siderophore moiety, enabling it to exploit iron transport systems for enhanced uptake into GNB [12,13]. CZA is a novel β-lactam/β-lactamase inhibitor combination active against Classes A and C, and some Class D β-lactamases [14]. IMR and MPV are active against Classes A and C β-lactamases [15]. ERV, a fully synthetic fluorocycline, is active against carbapenem-resistant Enterobacterales (CRE). PLZ, a next-generation aminoglycoside, imparts significant resistance to degradation by aminoglycoside-modifying enzymes (AMEs) [16].

Combining available antimicrobials often restores activity against resistant strains by leveraging synergistic pharmacodynamic interactions. Combinations of aztreonam (AZT) with CZA, MEM + fosfomycin (FOS), and amikacin (AK) + FOS have demonstrated promising in vitro synergistic effects [8,17]. The synergistic effect between AZT and avibactam positions this combination as a promising therapeutic option for infections caused by MBL-producing strains [18,19]. AZT has intrinsic activity against MBLs but is hydrolysed by extended-spectrum β-lactamase- and AmpC-producing organisms [20]. Avibactam, in turn, inhibits Classes A and C, and some Class D β-lactamases. In the absence of the AZT/avibactam combination, the AZT-CZA combination is a good alternative [17,21]. The MEM + FOS demonstrated significant synergy against CRKP strains expressing NDM, KPC, and OXA-48 [8,22,23]. AK + FOS has been reported as an alternative to colistin against OXA-48 and select KPC variants [8,24].

This study explored the molecular epidemiology, resistance evolution, and potential treatment strategies for high-risk CRKP lineages. We analysed the genomic landscape of CRKP, assessed the susceptibility profile to the new antimicrobials, and investigated antimicrobial synergy in CZA + AZT, MEM + FOS and AK + FOS combinations.

## 2. Results

**Figure 1:**
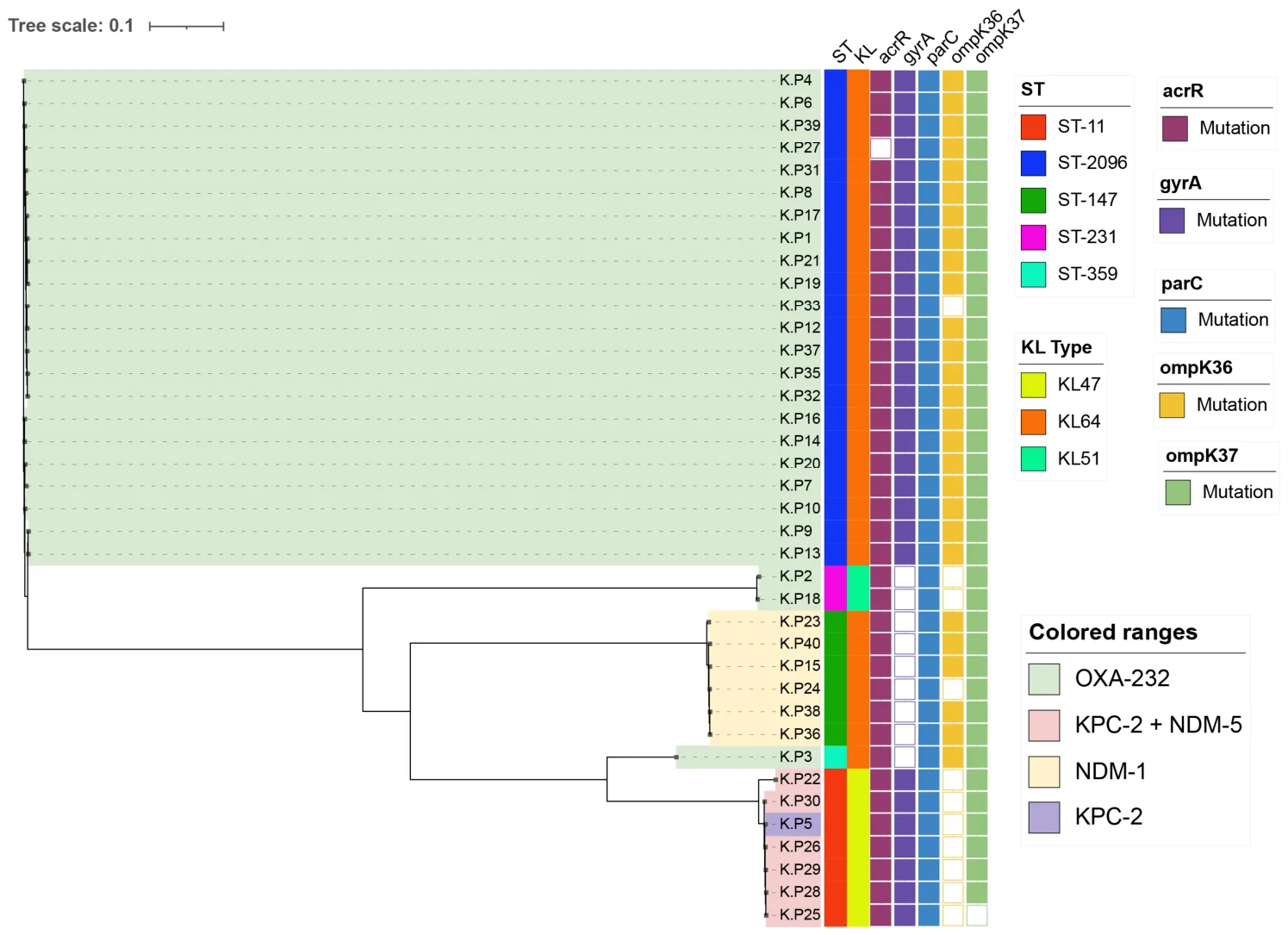
Phylogenetic Tree of CRKP based on whole-genome sequencing. Annotation tracks indicate sequence type (ST), capsular locus (KL) type, and the presence of mutations in acrR, gyrA, parC, ompK36, and ompK37. Shaded backgrounds highlight isolates carrying different carbapenemase genotypes (OXA-232, NDM-1, KPC-2, and KPC-2 + NDM-5). The tree was visualised and annotated using iTOL.

This study on genomic characterisation, activity of newly introduced antibiotics and in vitro synergy yielded thought-provoking results. The CRKP were isolated from urine, blood, respiratory, peritoneal, abdominal and wound samples. Genomic characterisation of 38 CRKP yielded 25 OXA-232, 6 NDM-5, 6 NDM-1 + KPC-2 and 1 KPC-2, revealing that XDR OXA-232 multilocus sequence typing (MLST)-2096 predominated (88%, 22/25), followed by ST-231 (*n* = 2) and ST-359 (*n* = 1) (Figure 1). All NDM-5 were ST-147 and NDM-1 + KPC-2 were ST-11. Capsular typing revealed a distinct pattern, with all OXA-232, ST-2096 and NDM-5, ST-147 being characterised by KL-64, KPC-2 + NDM-5 by KL-47 and OXA-232, ST-231 by KL-51 (Figure 1). They displayed either XDR or pan-drug resistant (PDR) profile. Isolate-wise distribution of carbapenemases, MLST and infection source is provided in Table 1.

### 2.1. Antimicrobial Resistance Genes

**Figure 2:**
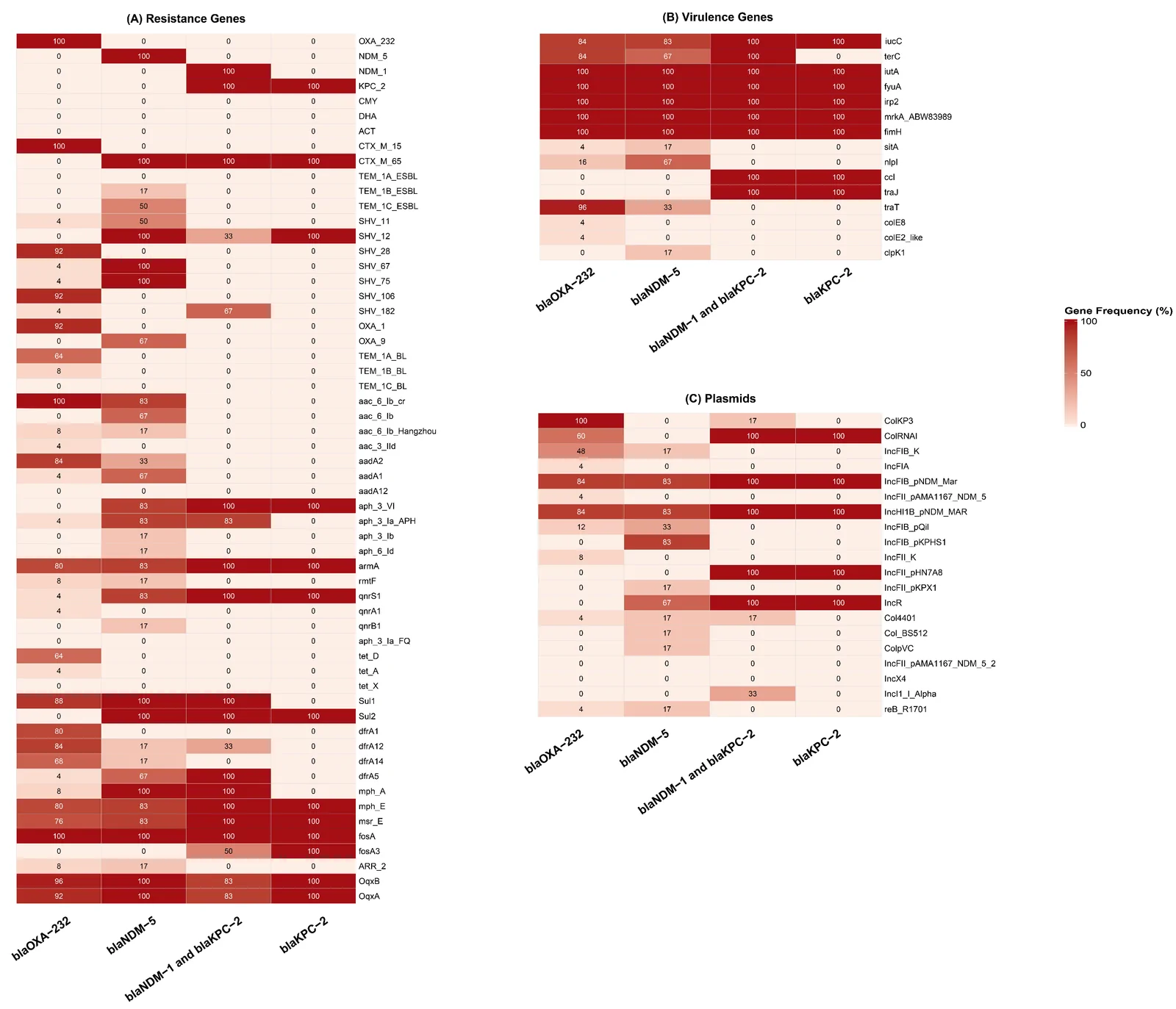
Heatmap of resistance, virulence and plasmid genes of carbapenem-resistant *K. pneumoniae*. The heatmap illustrates the percentage (%) of isolates carrying targeted genetic markers stratified into four core carbapenemase backgrounds (OXA-232, NDM-5, NDM-1 and KPC-2, and KPC-2). Cell frequencies range from light pink (0% prevalence) to red (100% prevalence). (**A**) Resistance genes, (**B**) virulence genes and (**C**) plasmid genes are depicted in the heatmap against the three Ambler classes.

A plethora of AMR genes were observed across all the carbapenemases (Figure 2A).

**Figure 3:**
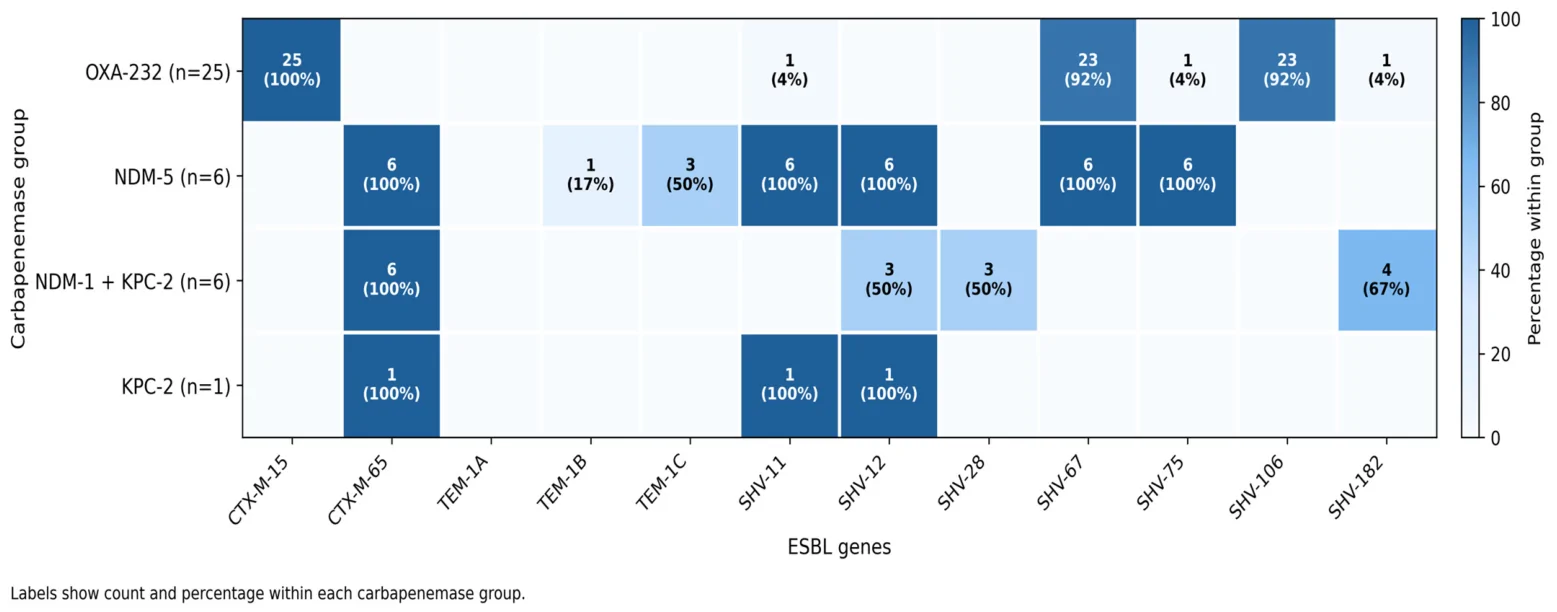
ESBL distribution in specific carbapenemases in OXA-232, NDM-5, NDM-1 + KPC-2, and KPC-2 is shown.

A varied and distinct burden of ESBLs was carried by the different CPEs, amongst which CTX-M-15, SHV-75 and SHV-12 (62.5%, 26/38) predominated, as seen in Figure 3. Others were CTX-M-65 (32.5%, 13/38), SHV-67 (22.5%, 9/38), SHV-11 (22.5%, 9/40), and SHV-28 (17.5%, 7/40) followed by SHV-106 (12.5%, 5/40), TEM-1C (10%, 4/40), TEM-1B (7.5%, 3/40), and TEM-1A (2.5%, 1/40).

OXA-232 exclusively carried CTX-M-15 (100%, 25/25), while all NDM/KPC isolates carried CTX-M-65 (12/12, 100%). Other ESBLs carried by OXA-232 were SHV-28 (92%, 23/25), and SHV-106 (100%, 25/25). NDM-5 isolates exhibited the most extensive ESBL spectra, with all strains (6/6) carrying CTX-M-65, SHV-11, SHV-12, SHV-67, and SHV-75. In contrast, NDM-1 + KPC-2 co-harbouring isolates exhibited narrower spectrum (CTX-M-65, TEM-1B, TEM-1C, SHV-11, and SHV-28). The KPC-2 isolate carried CTX-M-65, SHV-11 and SHV-12. Remarkably, no Ambler class C β-lactamase (AmpC) genes (DHA, ACT or CMY) were identified in this study.

Alarmingly, the most frequently detected aminoglycoside resistance determinants were *armA* (86.8%, 33/38) and *aac(6′)-Ib-cr* (78.9%, 30/38), followed by *aadA2* (65.8%, 25/38), *aph(3′)-Ia* (34.2%, 13/38), *aph(3′)-VI* (31.6%, 12/38), *aadA1* (15.8%, 6/38), *aac(6′)-Ib-Hangzhou* (13.2%, 5/38), *rmtF* (13.2%, 5/38), *aac(6′)-Ib* (10.5%, 4/38), and *aac(6′)-IId* (5.3%, 2/38). The least frequently detected determinants were *aph(3″)-Ib* and *aph(6)-Id*, each present in 2.6% (1/38) of isolates. Notably, *armA* was widely distributed across the major carbapenemase-producing groups, supporting the presence of high-level aminoglycoside resistance among these CRKP isolates.

OXA-232-producing isolates were characterised by the predominant carriage of *armA*, *aac(6′)-Ib-cr*, and *aadA2*, with less frequent detection of *aac(6′)-Ib-Hangzhou*, *rmtF*, and *aac(6′)-IId*. NDM-5-producing isolates demonstrated a broader aminoglycoside resistance profile. Apart from *armA* and *aac(6′)-Ib-cr*, these isolates harboured diverse aminoglycoside-modifying enzymes including *aadA2*, *aph(3′)-Ia*, and *aph(3′)-VI*. Sporadic detection of *aac(6′)-Ib-Hangzhou* and *rmtF* was also observed. Isolates co-harbouring NDM-1 + KPC-2 carried predominantly *armA*, *aph(3′)-VI* and *aph(3′)-Ia*.

The fluoroquinolone-modifying enzyme *aac(6′)-Ib-cr* predominated (78.9%; 30/38), followed by *qnrB1* (34.2%; 13/38) and *qnrA1* (13.2%, 5/38). OXA-232-producing isolates predominantly harboured *aac(6′)-Ib-cr*, with occasional carriage of *qnrB1* and *qnrA1*. In contrast, several NDM-5-producing isolates co-harboured *aac(6′)-Ib-cr*, *qnrB1*, and *qnrA1*. Isolates co-harbouring NDM-1 and KPC-2 also carried *aac(6′)-Ib-cr* together with sporadic presence of *qnr* genes.

Sulphonamide resistance genes showed a distinct distribution across the three CPE groups. Among OXA-232-producing isolates, only *sul1* was detected (88%, 22/25), whereas all NDM-5- and NDM-1 + KPC-2 producing isolates carried both *sul1* and *sul2* (100%, 6/6 each). Tetracycline resistance genes were exclusively observed among OXA-232 producers, with the *tet(D)* gene being present in 68% (17/25) of OXA-232 isolates, and *tet(A)* in 4% (1/25). In contrast, neither *tet(D)* nor *tet(A)* were detected among NDM-5-and NDM-1 + KPC-2-producing isolates.

Fosfomycin resistance was highly conserved across the major CPE groups. The *fosA* gene was detected in all OXA-232-producing isolates (100%, 25/25), all NDM-5-producing isolates (100%, 6/6), and all NDM + KPC co-producing isolates (100%, 6/6). In contrast, *fosA3* was present in 50% (3/6) of NDM + KPC co-producing isolates only.

Mutations were detected in five chromosomal loci: *ompK36*, *ompK37*, *acrR*, *gyrA*, and *parC* (Supplementary Table S1). The fluoroquinolone-resistance-associated mutation parC p.S80I was present in all isolates, while *gyrA* p.D87G was detected in 29/38 isolates. In addition, 37/38 isolates carried the same *acrR* mutation profile, comprising p.P161R, p.G164A, p.F172S, p.R173G, p.L195V, p.F197I and p.K201M. Mutations in *ompK36*, *ompK37* and *acrR* were detected in 37/38 isolates 97.4% (Figure 1). The *ompK36* mutation profile was clustered into four main patterns. The dominant profile was detected in 21/38 isolates and included multiple substitutions together with predicted frameshift-associated changes, namely p.N49S, p.L59V, p.A190W frameshift, p.L191S, p.F207W, p.D224E, p.Q227S frameshift, p.L228V, p.E232R, p.T254S, p.N304E frameshift, p.A217S, and p.N218H. A second profile, characterised by *ompK36* p.N49S, p.L59V, and p.T184P, was observed in 9/38 isolates. A third profile, comprising p.N49S, p.L59V, p.G189T, p.F198Y, p.F207Y, p.T222L, p.D223G, p.Q227S frameshift, p.L228K frame restoration, p.E232R, p.N304E, and p.A217S, was detected in 7/38 isolates. One isolate carried only p.A217S and p.N218H. Across all isolates, the most frequent *ompK36* substitutions were p.N49S and p.L59V, each detected in 37/38 isolates, followed by p.A217S in 29/38 isolates, p.Q227S frameshift and p.E232R in 28/38 isolates each, and p.N218H in 22/38 isolates. For ompK37, p.I70M and p.I128M were detected in 37/38 isolates. Among these, nine isolates additionally carried p.N230G and p.M233Q frameshifts. Additionally, *oqxA* and *oqxB* were detected in (35/38, 92.1%) and (36/38, 94.74%) isolates, respectively, while *acrR* mutations were near-global. Point mutations (P161R, G164A, F172S, R173G, L195V, F197I, K201M) in the acrR gene were present in (37/38, 97.37%) isolates.

### 2.2. Distribution of Virulence Genes

Whole-genome analysis of CRKP isolates revealed the presence of multiple virulence-associated genes, with varying prevalence across isolates producing OXA-232, NDM-5, NDM-1 + KPC-2 and KPC-2 (Figure 2B). All OXA-232, NDM-5, NDM-1 + KPC-2 and KPC-2 producing isolates carried ferric aerobactin receptor (*iutA*), siderophore receptor (*fyuA*), high molecular weight protein synthetase (*irp2*), type 1 fimbrial adhesin (*fimH*), and the non-ribosomal peptide synthetase gene *mrkA:ABW83989*.

**Figure 4:**
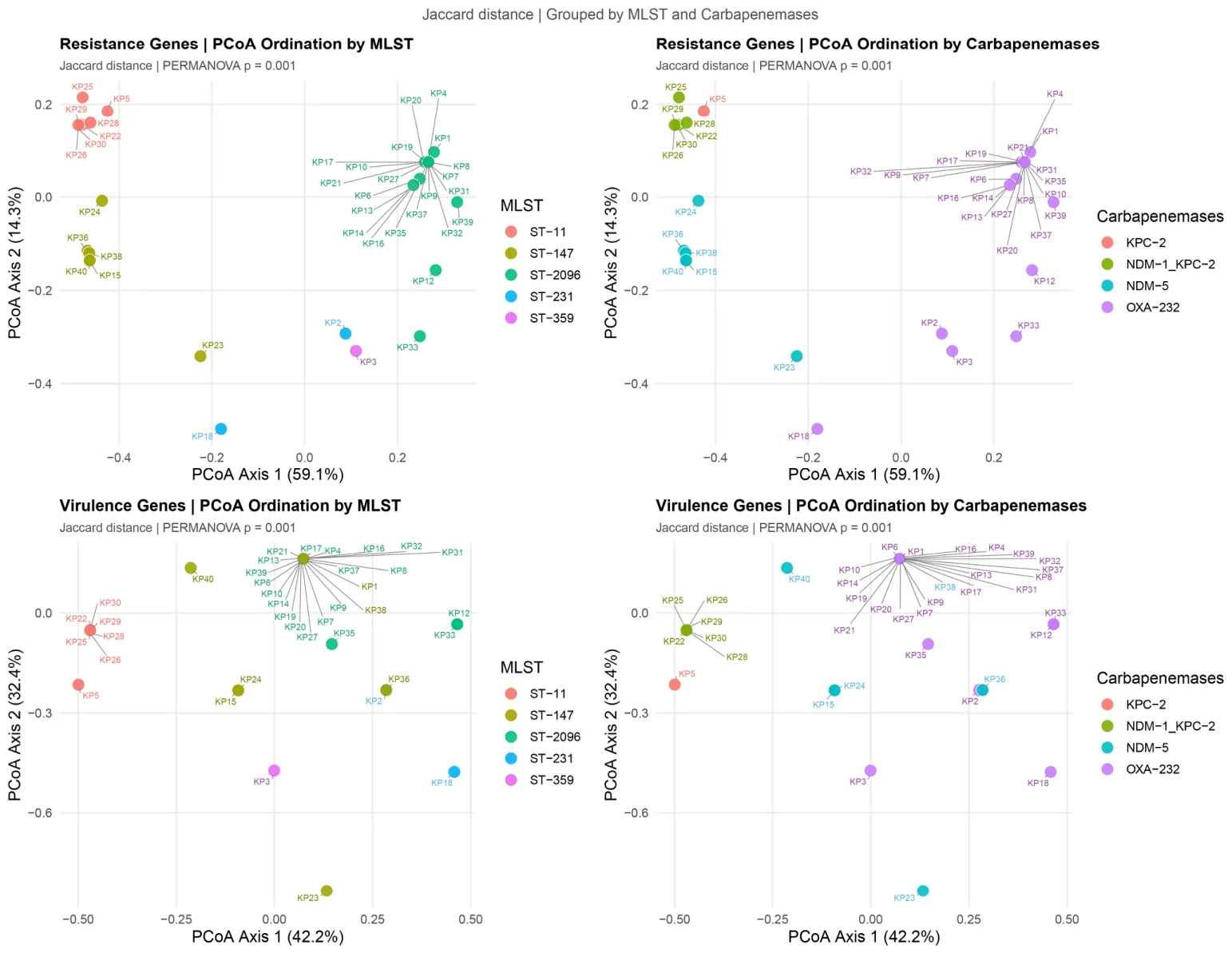
Clustering of resistance and virulence genes in MLST and carbapenemases. Each point represents one isolate, labelled by strain ID; isolates with identical profiles are collapsed to a single point with the constituent strains listed alongside.

Apart from these, OXA-232-producing isolates carried the outer membrane complement resistance gene *traT* (96%), aerobactin synthetase *iucC*, tellurium resistance gene *terC* (84%, 21/25), *sitA* (1/25), and *NlpI* in 1/25 isolates. Colicin-related genes (*colE8*, *colE2*-like) were each identified in 4% of isolates. In NDM-5 producers, *iucC*, *terC*, and *NlpI* were detected in (83.3%, 5/6), (66.7%, 4/6), and (66.7%, 4/6) isolates, respectively. In contrast, *traT* was detected in 2/6 isolates, and the iron transport gene *sitA* was identified in a single strain (1/6). The rare *clpK1* gene was identified in one isolate (16.7%, 1/6). Isolates co-carrying NDM-1 + KPC-2 additionally possessed *iucC*, *terC*, *ccI* and *traJ*. PCoA based on Jaccard distances demonstrated significant clustering of both resistance and virulence gene repertoires according to MLST and carbapenemase type (Figure 4).

### 2.3. Distribution of Plasmids

**Figure 5:**
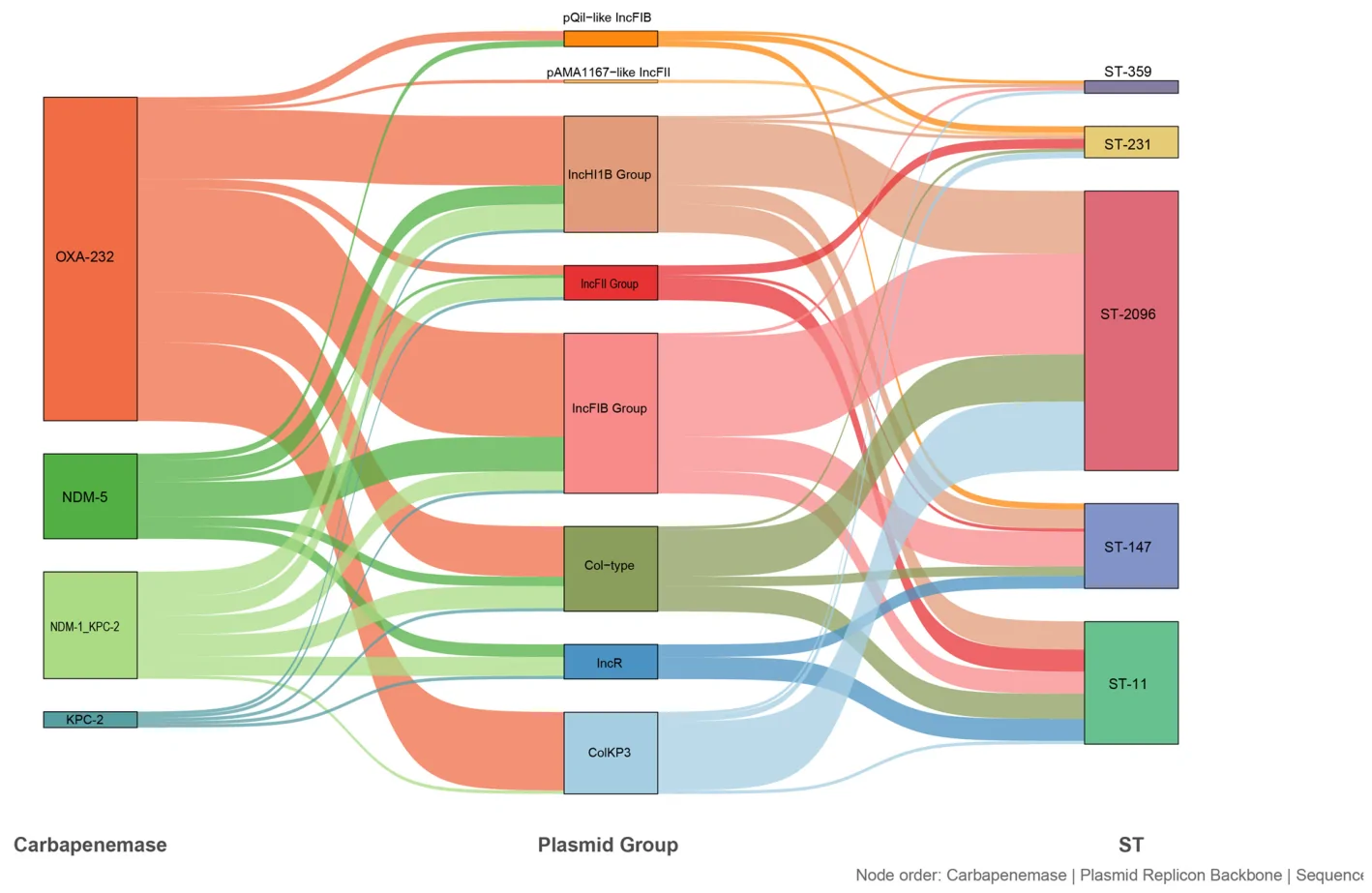
Sankey diagram illustrating the movement of plasmids within the CRKP and MLST. Carbapenemase (left) flow through plasmid replicon groups and terminates at respective MLST’s. Plasmid replicons were consolidated into operational nodes based on sequence identity, incompatibility classification, and size metrics. ColKP3 (OXA-232 vector): maintained as an independent node; represents a highly conserved, mini-replicon (<7 kb) exhibiting rigid vertical inheritance. pAMA1167-like IncFII: collapsed structural variants IncFII_pAMA1167_NDM_5 and _5_2 into a single conjugative epidemic framework. pQil-like IncFIB: maintained independently as a globally recognised, high-risk multi-replicon KPC-driving scaffold. IncR Vector: maintained independently as a stable, multidrug-resistant, non-conjugative integrative element. IncFIB Group/IncFII Group: aggregated by traditional incompatibility family groups (e.g., IncFIB_K, _PNDM_Mar, _pKPHS1 and IncFII_K, _pHN7A8, _pKPX1, IncFIA) representing typical large *K. pneumoniae* virulence/MDR companion backbones. Col-type: consolidated small, mobilisable elements (<10 kb; ColRNAI, Col4401) acting as structural background variations. IncHI1B Group: aggregated rare or low-frequency heavy-metal megaplasmids (IncHI1B_PNDM_MAR) and atypical replicons (IncI1_1_Alpha, reB_R1701) to isolate primary epidemiological drivers.

Amongst 19 identified plasmids, (Figure 2C) IncFIB(pNDM-Mar) and IncHI1B(pNDM-Mar) predominated (89.4%, 34/38). ColKP3 and ColRNAI followed, (68.4%, 26/38) and (63.1%, 24/38), respectively, with IncFIB(K) identified in 36.8%, 14/38 isolates. Less commonly detected plasmid types included IncFII(pHN7A8) (17.5%, *n* = 7), IncFIB(pQil) (15%, 6/40), and IncFIB(pKPHS1) (12.5%, 5/40). Percentages of other identified plasmids (IncFII(K), Col440I, Col(BS512), ColpVC, IncFIA, IncFII(pKPX1), and IncFII(pAMA1167-NDM-5) were lower. The Sankey analysis (Figure 5) demonstrated a complex but structured relationship between carbapenemase type, plasmid replicon backbone and sequence type. The distribution of plasmid replicons suggests potential routes for carbapenemase dissemination across the major clonal backgrounds. OXA-232-producing isolates showed the broadest plasmid associations, particularly with ColKP3, IncFIB and IncHI1B backbones, with these plasmid profiles converging predominantly within ST-2096. NDM-5 and NDM-1/KPC-2 co-producing isolates were likewise associated with IncFIB-, IncHI1B- and Col-type replicons, but were distributed exclusively across ST-11 and ST-147. The largest downstream convergence was observed in ST-2096, indicating that this sequence type was the principal clonal background associated with OXA-232 and diverse plasmid carriage. ST-147 and ST-11 also showed links with several plasmid groups, suggesting additional high-risk lineages with heterogeneous plasmid content. In contrast, *NDM-5*, *NDM-1*/*KPC-2* co-carriage and *KPC-2* alone contributed smaller flows.

**Figure 6:**
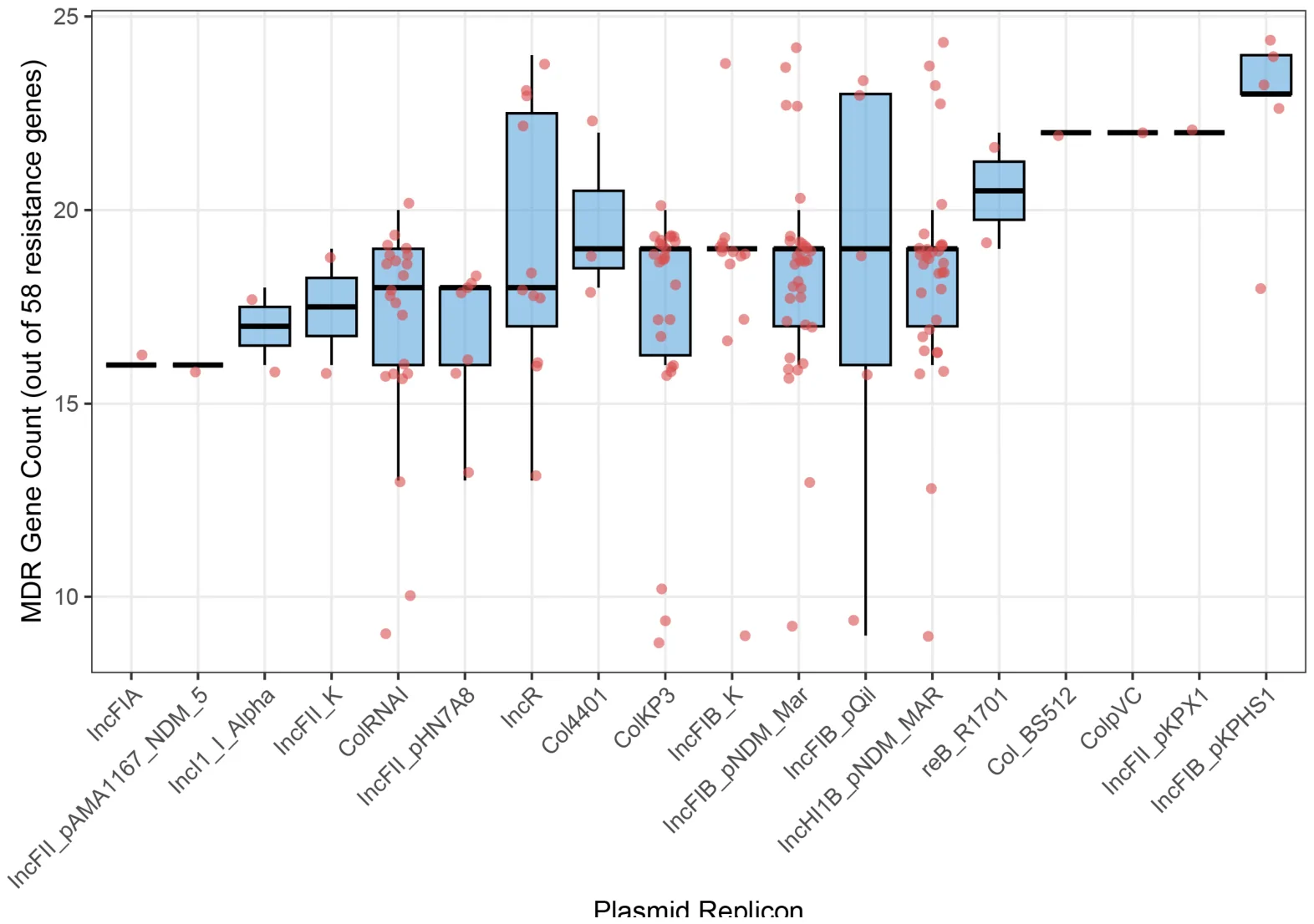
AMR gene load in plasmid replicons. Replicons are ordered left to right by ascending median resistance gene load. Each red dot represents a bacterial isolate. An isolate may carry more than one replicon and therefore contribute to more than one box.

Figure 6 showcases plasmid-wise estimated AMR gene load. Among OXA-232 isolates, ColKP3 was present uniformly (100%, 25/25), followed by IncFIB(pNDM-Mar) and IncHI1B(pNDM-MAR), (84%, 21/25) each. ColRNAI was present in (60%, 15/25) while IncFIB(K) at (48%, 12/25), and IncFIB(pQil) at (12%, 3/25), IncFII(pHN7A8) (8%, 2/25), Col440I (4%, 1/25), and Col (BS512) (4%, 1/25) were infrequent. In NDM-5 isolates, a distinct plasmidome was noted. IncFIB(pNDM-Mar), IncHI1B(pNDM-MAR) and IncFIB(pKPHS1) were the most prevalent plasmid-type replicons, 83.3% or 5/6 each, followed by IncR (66.7%, 4/6) and IncFIB(pQil) (33.3%, 2/6). IncFII(pKPX1), Col4401, Col(BS512), ColpVC and reB(R1701) were identified in one isolate only. The isolates co-carrying NDM-1 + KPC-2 revealed the highest diversity in plasmid type, with multiple replicons detected in all isolates. The plasmid types ColRNAI, IncFIB(pNDM-Mar), IncHI1B(pNDM-MAR), IncFII(pHN7A8), and IncR were consistently detected across all isolates (6/6). Isolate-wise distribution of plasmids, MGE, AMR, and virulence genes is provided in Supplementary Table S2.

The ST-2096/OXA-232 isolates formed a dominant cluster with a conserved resistance gene profile, while ST-11/NDM-1_KPC-2 and ST-147/NDM-5 isolates occupied distinct ordination spaces. Virulence gene profiles were also significantly structured, although with greater within-group dispersion, suggesting that resistance determinants are more tightly linked to clonal background whereas virulence genes show additional accessory genome variability.

Analysis of mobile genetic elements (MGEs) revealed substantial variability among the major carbapenemase-producing CRKP lineages (Table 2). OXA-232-producing isolates demonstrated moderate MGE burdens, ranging from 10 to 24 MGEs per isolate. In contrast, NDM-5-producing isolates exhibited greater variability, with MGE counts ranging from 11 to 29 per isolate. The highest MGE burden was observed among NDM-1 + KPC-2 co-producing isolates, with counts ranging from 25 to 36 MGEs per isolate. Overall, isolates harbouring NDM-1 + KPC-2 demonstrated consistently higher MGE content than OXA-232-producing isolates.

### 2.4. Susceptibility of CRKP to Newer Antimicrobial Agents

A total of 135 (72 OXA-48-like, of which 25 were OXA-232; 40 NDM-producing isolates, of which 6 were NDM-5; 22 KPC-producing isolates and 6 with dual carriage of NDM-1 + KPC-2) were tested.

Amongst the nine new antimicrobials tested, CFD demonstrated the greatest overall activity across all carbapenemase groups (Table 2). The MIC50/MIC90 of these antimicrobials are seen in Table 3. Among OXA-48-like carbapenemases, including OXA-232, MIC50/MIC90 values were 0.125/0.5 µg/mL. Similarly low MIC values were observed among NDM-producing isolates (0.125/0.5 µg/mL) and NDM-5-producing isolates (0.064/0.5 µg/mL). Activity remained favourable against both KPC and NDM-1 + KPC-2 co-producing isolates (0.5/1 µg/mL).

CZA demonstrated 625% susceptibility overall. It exhibited potent activity against OXA-48-like and OXA-232 with MIC50/MIC90 values of 1/2 µg/mL (Table 3). KPC-producing isolates demonstrated 1/4 µg/mL. Poor activity was observed against NDM-producing isolates, with MIC50/MIC90 being ≥256 µg/mL. ERV displayed consistently potent activity across all carbapenemase groups. MIC50 values ranged from 0.25 to 0.5 µg/mL, while MIC90 values ranged from 0.5 to 1 µg/mL. The lowest MIC50 value was observed among KPC-producing isolates (0.25 µg/mL), whereas OXA-48-like, OXA-232, NDM-5, and NDM-1 + KPC-2-producing isolates all demonstrated MIC50 values of 0.5 µg/mL. FOS exhibited variable activity across the carbapenemase groups. OXA-48-like and OXA-232-producing isolates demonstrated MIC50 values of 16 µg/mL; however, MIC90 values exceeded 256 µg/mL. NDM-5-, KPC-, and NDM-1 + KPC-2-producing isolates showed markedly elevated MIC50 and MIC90 values ≥ 256 µg/mL. PLZ demonstrated alarmingly limited activity with 42% susceptibility. OXA-48-like and OXA-232-producing isolates showed MIC50 values of 64 µg/mL with MIC90 values exceeding 256 µg/mL. NDM-producing isolates similarly demonstrated poor susceptibility (MIC50/MIC90 ≥ 256/≥256 µg/mL). Although NDM-1 + KPC-2-producing isolates exhibited a lower MIC50 of 2 µg/mL, the MIC90 remained >256 µg/mL. IMR and MVP demonstrated good activity against KPC-producing isolates, with MIC50/MIC90 being 0.5/2 µg/mL.

### 2.5. In Vitro Evaluation of Antimicrobial Synergy Against CRKP

The synergistic activity of MER + FOS and AK + FOS was evaluated against OXA-232, NDM-5-, KPC-2-, and NDM-1 + KPC-2, while CZA + AZT was evaluated against NDM-5-, KPC-2-, and NDM-1 + KPC-2-producing CRKP using the gradient diffusion cross method and interpreted according to the fractional inhibitory concentration index (FICI). The results are described in Table 4.

**Figure 7:**
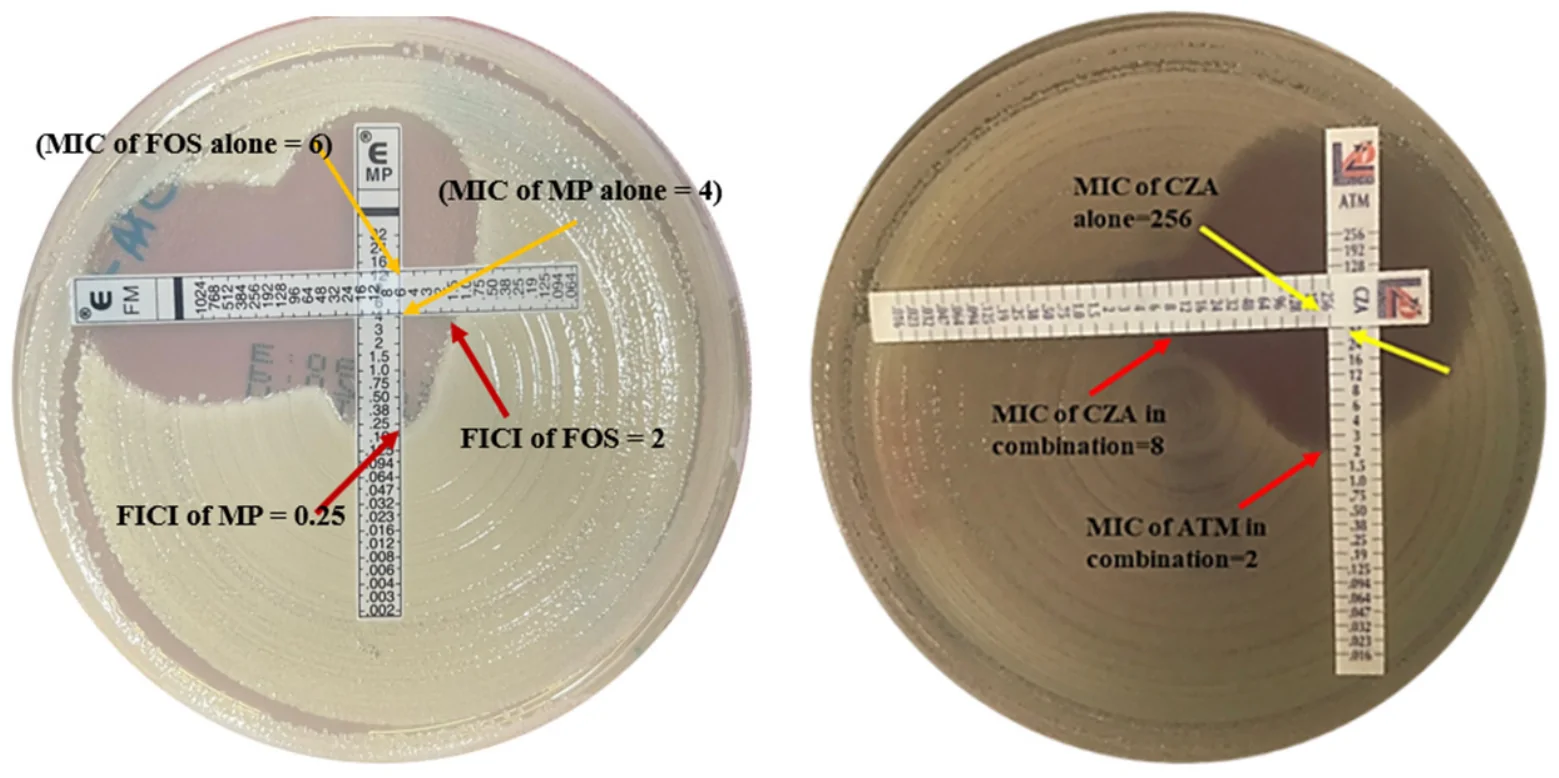
Antimicrobial synergy testing by gradient diffusion cross method.

The MER + FOS combination demonstrated variable activity depending on the underlying carbapenemase (Figure 7). Among OXA-232-producing isolates, synergy was observed in 32% (8/25), partial synergy in 16% (4/25), and additive activity in 8% (2/25), whereas 44% (11/25) of isolates demonstrated indifferent interactions. In contrast, no synergistic activity was observed among NDM-5- or NDM-1 + KPC-2-producing isolates. Partial synergy was detected in only 16.7% (1/6) of isolates in each group, while the majority demonstrated indifferent interactions (83.3%, 5/6). The single KPC-2-producing isolate also demonstrated an indifferent response. No antagonism was observed for any isolate.

The AK + FOS combination showed moderate activity against OXA-232-producing isolates, with synergy observed in 40% (10/25), partial synergy in 12% (3/25), and additive activity in 4% (1/25) of isolates. However, 44% (11/25) demonstrated indifferent interactions. No synergy was observed among NDM-5-producing isolates, all of which demonstrated indifferent responses. Among NDM-1 + KPC-2-producing isolates, synergy was observed in 16.7% (1/6), additive activity in 33.3% (2/6), and indifference in 50% (3/6) of isolates. The single KPC-2-producing isolate again demonstrated an indifferent interaction. No antagonistic effects were identified with any antimicrobial combination tested. Among OXA-232-producing isolates, AK + FOS and MER + FOS demonstrated overall synergy/partial synergy rates of 52% and 48%, respectively.

The CZA + AZT combination demonstrated 100% synergistic activity among all combinations tested. Synergy was observed in all OXA-48-like (25/25) and (6/6) of NDM-5-producing (6/6), NDM-1 + KPC-2 co-producing (6/6), and the single KPC-2-producing isolate.

## 3. Discussion

Distinct associations between carbapenemase type, MLST, resistome, virulome, and plasmidome were observed amongst the CRKP. Our study revealed a predominance of OXA-232, ST-2096, a high-risk derivative of the globally disseminated ST-14 clonal complex, prevalent in the Middle East and India, and the emergence of NDM-5 and NDM-1 + KPC-2 [25,26]. The prominent association of OXA-232 with multiple plasmid groups and its strong convergence within ST-2096 highlights the potential role of this clone as an important regional vehicle for the spread of carbapenem resistance [27]. This was a dramatic change from our previous study where ST-231 predominated and ST-147 had emerged recently [8]. The emergence of NDM-5 and dual NDM-1 + KPC-2-producing CRKP represents a critical public health threat in the Middle East. Driven by transmissible plasmids, these multidrug-resistant isolates severely limit therapeutic options and have been linked to high mortality rates in intensive care units across the region [27]. A multicentric study from Türkiye also reports a shift from ST-2096 OXA-232 to co-carriage of NDM-5 and OXA-48 in ST-147 over a five-year period [28]. Both ST-147 and ST-11 are internationally recognised high-risk CRKP clones frequently associated with dissemination of carbapenemases and multidrug resistance plasmids [29,30,31]. ST-147 has emerged globally as an important carrier of *blaNDM* variants and is frequently associated with extensive genomic plasticity and plasmid-mediated resistance acquisition [32]. Therapeutically challenging, the coexistence of NDM and KPC carbapenemases within ST-11 is increasingly being reported [33,34]. As observed in this study, a predominance of KL-64 in OXA-232, ST-2096 and NDM-5; ST-147 and KL-47 in NDM-1 + KPC-2 ST-11; and ST-147 KL-51 in ST-231 is being increasingly reported in high-risk hospital-associated CRKP clones [5,28,35]. A large multicentre longitudinal study from China reported that KL64 and KL47 were the dominant CRKP capsular types [36]. Some evidence is emerging that KL64 may have evolved from an ST11-KL47-like ancestor through capsular recombination [37]. KL-51 in NDM-1 + KPC-2, ST-11 has been reported among CRKP, in association with ST23 previously [26].

OXA-232 were characterised by *blaCTX-M-15*, *blaSHV-28*, *blaSHV-106*, *armA*, *aac(6′)-Ib-cr*, *fosA*, and multiple *dfrA* and *sul* genes, consistent with previous reports [38]. Compared with OXA-232-, the NDM-5 group carried a broader array of AMR genes, including a larger ESBL repertoire (*blaCTX-M-65*, *blaSHV-11*, *blaSHV-12*, *blaSHV-67*, and *blaSHV-75)*, and *armA*, *aac(6′)-Ib-cr*, and *fosA*, among others, supporting ongoing horizontal acquisition of accessory resistance genes [39]. The NDM-1 + KPC-2-producing ST-11 isolates demonstrated the most complex resistome architecture, with simultaneous carriage of *blaNDM-1*, *blaKPC-2*, *blaCTX-M-65*, *armA*, *fosA3*, and *qnr* genes.

As expected, *aac(6′)-Ib-cr* was widespread, highlighting the extensive dissemination of plasmid-mediated amikacin and fluoroquinolone resistance. The consistent presence of 16S rRNA methyltransferase genes—*armA* in most and *rmtF* in others across all but one 38 CPE (OXA-232, NDM-5, and NDM-1/KPC-2)—is a grave public health concern [40]. This development has been unexpectedly rapid in Oman [8]. These enzymes confer pan-aminoglycoside resistance, PLZ including by modifying the aminoglycoside target site, corroborated by PLZ’s unexpectedly poor activity (41%) in our study. Similar reductions in PLZ susceptibility have been reported among CRKP populations carrying *armA* and *rmt* methyltransferase genes [41].

The universal presence of fosA among all the CRKP and fosA3 in the NDM + KPC indicates active acquisition of mobile fosfomycin resistance determinants over the years. A previous study from our group had reported a far lower resistance [8]. FOS demonstrated limited activity, with high MIC50/90 demonstrated against all CRKP. Similar associations between fosA carriage and elevated FOS MICs have been described globally [42]. It must, however, be remembered that though *armA*, *rmtF* and *fosA*, *fosA3*, were widespread, transcription was limited, as 28.6% and 41% susceptibility was observed in the CRKP.

Sulphonamide resistance genes differed by CRKP type, with OXA-232 isolates associated with *sul1*, while NDM-5 and NDM + KPC carried both *sul1* and *sul2*. Tetracycline resistance determinants were largely restricted to the OXA-232-producing lineage in this cohort, with 68% carrying the *tet(D)* gene. ERV exhibited consistently low MIC50 and MIC90 values across all carbapenemase groups, confirming its broad activity against CRKP [43]. As a fully synthetic fluorocycline, ERV retains activity despite the presence of *tet(D)* and efflux pumps. ERV may be considered a valuable carbapenem-sparing option for management of CRKP infections.

The dominant ompK36 profile included multiple substitutions and predicted frameshift-associated changes further contributing to the CRKP phenotype. Structural and functional studies have shown that a GD insertion in OmpK36 loop 3 constricts the pore, restricts carbapenem diffusion and increases meropenem MICs in a KPC-producing background [44]. Point mutations in genes encoding OmpK35 and OmpK36 have contributed to the emergence of CRKP [45]. Persistent selective pressure resulting from extensive carbapenem use in hospital settings may promote the emergence and maintenance of these mutations, despite the potential fitness costs they impose on the bacteria [44]. Compared with OmpK36, the contribution of OmpK37 to carbapenem resistance remains less established [46]. While *parC* mutations mediating fluoroquinolone resistance were present across the CRKPs, dual carriage of *parC* and *gyrA* mutations was carried by OXA-232 ST-2096 and NDM-1 + KPC-2 ST-11 isolates [47]. The frequent detection of *acrR* and *parC* supports a broader multifactorial resistance to fluoroquinolones, involving both target-site mutations and potential efflux dysregulation.

Despite the plethora of AMR genes, excellent susceptibility was observed to CFD and CZA + AZT in all CRKP. CFD demonstrated the most consistent activity across all carbapenemase groups, consistent with other studies [48,49]. The unique siderophore-mediated mechanism of cefiderocol allows active transport into the bacterial periplasmic space via iron uptake systems, enabling it to overcome several resistance mechanisms, including MBL, porin loss, and efflux pump overexpression. CZA demonstrated potent activity against OXA-48-like, OXA-232, and KPC-producing isolates and should be considered an important therapeutic option for OXA-48-like CRKP infections, while MPV and IMR demonstrated favourable activity primarily against KPC-producing isolates [50]. For non-severe CRKP infections, older antimicrobial agents may still be considered according to the site of infection, while aminoglycosides, including PLZ, represent therapeutic options for complicated urinary tract infections [51].

The virulence profile demonstrated a conserved pathogenic backbone across all four CPE groups, with *iutA*, *fyuA*, *irp2*, *mrkA* and *fimH* present in 100% of isolates. The *fimH* and *mrkA* genes encoding type 1 and type 3 fimbriae, respectively, facilitate adhesion to epithelial surfaces, colonisation and biofilm formation across CPE groups on medical devices, enhancing persistence within hospital environments [52]. The siderophore-associated genes *iutA* and *iucC* are linked to the aerobactin system, while *fyuA* and *irp2* are components of the yersiniabactin system; these iron-acquisition systems can enhance bacterial survival in iron-restricted host environments and are important contributors to *K. pneumoniae* pathogenicity. They contribute significantly to invasive disease and bloodstream survival [53,54]. OXA-232-producing isolates showed a particularly broad accessory virulence profile, with high frequencies of *iucC* (84%), *terC* (84%) and *traT* (96%). The predominance of *traT*, which has been associated with serum resistance, together with siderophore and fimbrial determinants, may favour persistence during invasive infection [45]. The NDM-1/KPC-2 co-producing isolates had the most uniform profile for several major determinants, with 100% carriage of *iucC*, *terC*, *iutA*, *fyuA*, *irp2*, *mrkA* and *fimH*. This combination of efficient iron acquisition, adherence and environmental-stress tolerance may be particularly concerning when coupled with extensive antimicrobial resistance.

NDM-5 and NDM-1 + KPC-2 differed in carrying additional virulence genes: *sitA/sitABCD*, *clpK1*, *nlpI*, genes associated with oxidative stress resistance, heat tolerance, environmental persistence, and bacterial fitness in NDM-5 and *traJ*, *ccI*, and *terC* in NDM-1 + KPC-2 isolates. The conjugative transfer-associated gene *traJ* may facilitate plasmid mobilisation, while *terC* contributes to tellurite resistance and stress tolerance. Although classical hypervirulence-associated regulators such as *rmpA*, *rmpA2*, *iro*, and *clb* were not identified, the coexistence of siderophore-associated virulence genes suggests partial convergence of resistance and virulence among circulating CRKP lineages. The preservation of these core factors in carbapenemase-producing isolates suggests that resistance and virulence traits are co-maintained rather than mutually exclusive, echoing observations from global CRKP lineages in which successful clones retain both resistance and fitness determinants [55]. This study highlights that the widespread presence of conserved virulence determinants alongside extensive antimicrobial resistance suggests that the acquisition of carbapenemase genes has not compromised the pathogenic potential of these high-risk *Klebsiella pneumoniae* clones, thereby underscoring their clinical and epidemiological importance.

The plasmid and MGE distribution point to lineage-specific evolutionary patterns. The plasmid diversity indicates complex horizontal gene transfer mechanisms contributing to AMR. ColKP3 in OXA-232 are clearly linked with global dissemination [25,26]. The concurrent presence of IncFIB and IncHI1B replicons in all CPEs points to hybrid plasmids capable of maintaining XDR and virulence-associated genes [54].

The conserved plasmid distribution in OXA-232 is consistent with vertical transmission and long-term adaptation of plasmid–host combinations [56,57]. In contrast, NDM-5 were characterised with greater plasmid heterogeneity (IncR, IncFIB(pKPHS1), IncFII(pKPX1), Col440I, Col(BS512), and ColpVCcF). Their conjugative transfer capacities make them efficient vectors for *blaNDM* dissemination [58,59]. Non-conjugative IncR plasmids accumulate AMR cassettes through recombination with transposable elements and insertion sequences. The highest plasmid diversity was observed among NDM-1 + KPC-2-isolates, where multiple replicons including IncHI1B, IncFII(pHN7A8), IncR, and IncFIB(pNDM-Mar) coexisted together with high MGE counts. However, because replicon typing demonstrates plasmid carriage rather than the physical location of the carbapenemase gene, these associations should be interpreted as potential transmission pathways rather than definitive evidence of gene–plasmid linkage. It is noteworthy that pLVPK-like virulence plasmids that carry hypervirulence genes were missing in our cohort [31]. Elevated MGE burdens likely reflect intense recombination activity mediated by insertion sequences, transposons, and integrons, facilitating accumulation and mobilisation of resistance determinants including *blaNDM-1*, *blaKPC-2*, *armA*, and *fosA3* [60]. This raises significant clinical and epidemiological concerns, as they may serve as important reservoirs for dissemination of high-risk resistance plasmids [61]. The coexistence of aerobactin- and yersiniabactin-associated loci together with XDR determinants suggests partial convergence of virulence and resistance within these CRKP isolates.

The disturbingly low CFD (95%), ERV (74%), PLZ (41%), CZA (62%), IMR (43%), and MPV (30%) susceptibilities against CRKP reinforce the need to explore effective antimicrobial combinations with the available options. Combination antimicrobial regimens have the potential to restore activity against resistant strains by leveraging synergistic pharmacodynamic interactions, overcoming complex resistance mechanisms and improving treatment outcomes [8,62]. They should be considered when therapeutic options are restricted to polymyxins, aminoglycosides, tigecycline, or fosfomycin [51]. The present study evaluated combinations that are available in routine clinical practice, particularly in resource-limited settings where access to newer agents remains restricted.

This study provides important therapeutic insights for the management of infections caused by CRKP, particularly in settings where access to newer antimicrobial agents is limited. Among the antimicrobial combinations tested, CZA + AZT demonstrated the most potent in vitro activity, exhibiting 100% synergy against all OXA-232 (25/25), NDM-5 (6/6), NDM-1 + KPC-2 (6/6), and KPC-2-producing isolates. This combination relies on aztreonam’s resistance to MBL hydrolysis and avibactam’s inhibition of serine β-lactamases [63,64]. Khan et al., 2021 reported that the gradient diffusion method correlates well with the reference modified broth microdilution method [65]. Falcone et al., 2022 reported similar results with 60% reduction in risk of mortality, lower clinical failure, and a shorter length of hospital stay [66]. MEM + FOS and AK + FOS combinations demonstrated poor synergy, which contrasts with our prior report and aligns with the evolving AMR trends identified phenotypically and genotypically [8]. In this study, FOS–MEM demonstrated synergy in almost all of the tested XDR/PDR isolates, including OXA-232 producers, and substantially reduced MEM MICs in several isolates, while FOS–AK synergy was observed despite the presence of aminoglycoside and fosfomycin resistance determinants. Senegal et al., 2020 reported FOS + MEM synergy in 15/17 (88%), whereas FOS + AMK synergy in 29%. OXA-48 and NDM-producing *K. pneumoniae* bloodstream isolates [23]. However, another study reported lower synergy rates for FOS-MEM (20% of 50 CRKP isolates), despite 68% being OXA-48-like producers [67].

Both the type of CPE and MIC appear to impact the outcome. Among OXA-232 isolates, complete or partial synergy was observed in 48% and 52% isolates for MEM + FOS and AK + FOS, respectively. In contrast, NDM-5-and NDM-1 + KPC-2-producing isolates demonstrated indifferent interactions, with only occasional partial synergy. It was observed that OXA-232-producing isolates generally exhibited lower FOS and MEM MICs with subsequent greater reductions following combination testing, whereas in NDM-producing isolates, FOS and MEM MICs remained high despite combination exposure. Similarly, AK + FOS showed no synergism among NDM-5 isolates, characterised by high AK MICs, which remained unchanged after combination exposure. These findings suggest that, in NDM-5 isolates, high baseline MICs to MEM, FOS, and AK were associated with poor synergistic activity. A slightly better response was observed in NDM-1 + KPC-2 co-producing isolates, with one isolate demonstrating partial synergy with MEM + FOS. With AK + FOS, one isolate demonstrated synergy, two showed additive effects, and three remained indifferent. Our findings suggest that individual MICs may be an important determinant of synergy and may partially explain the superior performance of fosfomycin-containing combinations among OXA-232-producing isolates. Given its simplicity, low cost, and compatibility with routine laboratory workflows, the gradient diffusion cross method may offer a pragmatic approach for rapidly exploring potentially synergistic antimicrobial combinations against CRKP, particularly in settings where more complex synergy-testing methods are not readily available.

Although *fosA*, *fosA3*, *armA*, *rmtF* and related aminoglycoside resistance genes were widely distributed across the carbapenemase groups, their presence was not consistently associated with the loss of synergistic activity. This suggests that the genomic presence of resistance determinants may not fully predict phenotypic behaviour. Differences in gene expression, copy number, regulatory mechanisms, and enzyme activity may contribute to the observed variation in synergistic responses. Similar discrepancies between genotypic resistance markers and phenotypic susceptibility have been described previously for both FOS and AK [68].

## 4. Materials and Methods

This study was conducted from January 2021 to December 2024 at the Department of Microbiology and Immunology, College of Medicine and Health Sciences, Sultan Qaboos University and Hospital (SQU), in collaboration with the Central Public Health Laboratory (CPHL), Sultanate of Oman. The study was approved by the Medical Research Ethics Committee (MREC) in the College of Medicine & Health Sciences at SQU: REF. NO. SQU-EC/192/19.

Consecutive GNB from diverse clinical specimens (blood, 412; urine, 289; respiratory, 221; wounds/skin, 98; others, 35) were screened for CRKP and subjected to genotypic confirmation by Xpert Carba-R (Cepheid, Frankfurt, Germany). A total of 135 non-duplicate CRKP isolates from confirmed infectious disease cases were collected for further studies. Bacterial identification was confirmed using matrix-assisted laser desorption ionisation time-of-flight mass spectrometry (MALDI-TOF; Bruker, Bremen, Germany), while antimicrobial susceptibility testing was performed using the BD Phoenix automated system (Becton Dickinson Diagnostic Systems, Sparks, MD, USA). Interpretation of susceptibility results was based on the 2025 Clinical and Laboratory Standards Institute (CLSI) M100 guidelines (35th edition) [69].

### 4.1. Evaluation of Newer Antimicrobial Agents

The minimum inhibitory concentrations (MICs) of CFD, FOS, ERV, PLZ, minocycline (MIN), and aztreonam (AZT) were assessed against 135 CRKP by the gradient diffusion method. Ceftazidime–avibactam (CZA) was tested against OXA-48 and KPC isolates, while imipenem–relebactam (IMR) and meropenem–vaborbactam (MVP) were tested only against KPC producers. The strips for these agents were obtained from two manufacturers: Liofilchem (MTS), Liofilchem S.r.l., Via Scozia, Zona Industriale, Roseto degli Abruzzi (Italy) and bioMérieux (E-test) bioMérieux, Inc., Durham, NC, France). MIC50 and MIC90 values were calculated for each carbapenemase group separately. The test was performed as per the manufacturers’ instructions. Quality control strain *Klebsiella pneumoniae* (ATCC 700603) was utilised.

### 4.2. Whole-Genome Sequencing (WGS)

WGS was performed on a representative subset of 38 out of the 135 CRKP isolates to capture the genomic diversity of the study population while considering the substantial cost associated with WGS. The isolates reflected the diversity of carbapenemase genes and clinical specimen sources. The selected subset comprised all NDM-5-producing isolates (*n* = 6), all NDM-1/KPC-2 co-producing isolates (*n* = 6), the single KPC-2-producing isolate (*n* = 1), and a representative subset of OXA-232-producing isolates (*n* = 25), including both extensively drug-resistant (XDR) and pandrug-resistant (PDR) phenotypes. DNA was extracted from an overnight culture using the QIAamp DNA Mini Kit (Qiagen, Hilden, Germany) as per the manufacturer’s instructions, with minor modifications, as previously described [70]. Sequencing was performed at MicrobesNG (https://microbesng.com/, accessed on 18 July, 2023), Birmingham, UK, on the Illumina platform. Isolate processing was carried out using commercial extraction kits as per the manufacturer’s protocol. DNA libraries were prepared using standard Illumina library preparation protocols and subsequently sequenced on an Illumina next-generation sequencing platform to obtain paired-end reads. The resulting raw reads were subjected to quality assessment and trimming before being assembled de novo. Detailed resistance gene profiling, with bioinformatics analysis, was carried out using the Center for Genomic Epidemiology (CGE) website (https://www.genomicepidemiology.org/) accessed on 22 September 2023. Plasmid Finder and ResFinder, v.2.0 were used to identify plasmids and acquired antimicrobial resistance genes, respectively [71,72]. Additionally, the Comprehensive Antibiotic Resistance Database v3.2.8 (CARD) (https://card.mcmaster.ca/, accessed on 25 September 2023) was used to detect putative antimicrobial resistance genes using the Resistance Gene Identifier (RGI) tool v6.0.3. This tool identifies the antibiotic resistome(s) as well as point mutations within the resistance-conferring genes [73]. Capsular typing was performed from WGS FASTQ data using the *Klebsiella* PasteurMLST sequence definition database (https://bigsdb.pasteur.fr/cgi-bin/bigsdb/bigsdb.pl?db=pubmlst_klebsiella_seqdef). It was accessed on 16 June 2026 [74,75,76]. Chromosomal antimicrobial-resistance-associated mutations were analysed from WGS FASTQ data using the online ResFinder tool (https://genepi.food.dtu.dk/resfinder, 25 September 2023).

All the genome sequences were submitted to NCBI and accession numbers are provided in Supplementary Document S2. *K. pneumoniae* RJF293 (GenBank accession number CP014008, https://www.ncbi.nlm.nih.gov/nuccore/) [accessed on 2 February 2024] references were used. iTOL was used to visualise and annotate the tree.

### 4.3. Antimicrobial Synergy Testing

The thirty-eight WGS XDR/PDR CRKP were subjected to in vitro assessment of synergy of three antimicrobial combinations (CZA + AZT, MER + FOS, AK + FOS) by the gradient diffusion method, using Etest^®^ (bioMérieux, Inc., Durham, NC, USA) and Liofilchem (Liofilchem, S.r.l., Via Scozia, Zona Industriale, Roseto degli Abruzzi, Italy). The CZA + AZT combination was tested by three different methods: the gradient diffusion cross method, the gradient diffusion fixed ratio method, and the gradient diffusion MIC: MIC method [77]. The cross method was carried out as follows: The MIC of the individual antimicrobial agents was calculated as per CLSI guidelines [69]. The next day, 0.5 McFarland suspension of the test isolate was applied onto a Mueller–Hinton plate. The gradient diffusion strips of the selected antibiotic combination were carefully placed one over the other perpendicularly, so that they intersected at the MIC of the individual agents. Following overnight incubation, the new MIC values of each drug in combination were noted. The outcome of the combination was measured by calculating the fractional inhibitory concentration index (FICI) [77,78]: FICI = FICA + FICB, where FIC of agent A = MIC of agent A in combination/MIC of agent A alone. FIC of agent B = MIC of agent B in combination/MIC of agent B alone. The results were interpreted as follows: synergy, FICI ≥ 0.5; partial synergy, FICI < 0.5 to >1; addition, FICI ≥ 1 to ≤2; indifference, FICI ≥ 2 to 4; and antagonism = FICI > 4. Details of the E-test fixed ratio method and the MIC: MIC method tests are provided in the Supplementary Document S1. The results of all three methods were comparable (Supplementary Table S3). Due to the ease of performance, the other two combinations were assessed by the gradient diffusion method, using Etest^®^ (bioMérieux, Durham, NC, USA).

### 4.4. Statistical Tests

PCoA based on the Jaccard distance matrix of the binary gene-presence matrices was performed to examine clustering patterns of resistance and virulence gene repertoires by MLST and carbapenemase family. PERMANOVA was used to assess the genetic relatedness among strains and dissimilarities between groups. A clustered heatmap was generated in R using dplyr/tidyr to calculate the percentage of isolates carrying each gene within each carbapenemase group (OXA-232, NDM-5, NDM-1/KPC-2, KPC-2), visualised via ComplexHeatmap (prevalence %).

Boxplot for AMR gene load together with the replicons were generated via ggplot2 with overlaid jitter points in [79,80], with replicons ordered by ascending median MDR_Load, allowing for co-assessment of the central tendency, spread, and individual-level variation in resistance burden associated with each plasmid replicon type. Pathways consolidating carbapenemase genes, distinct multi-replicon plasmid combination profiles, and ST lineages were mapped sequentially through a multi-stage tracking framework implemented via the tidyverse package and ggsankey in RStudio (Version 2026.04.0+526) [79,80].

## 5. Conclusions

The structured analysis of MLST, resistome, and virulome of CRKP illustrates that these organisms combine extensive resistance with virulence attributes. WGS revealed that our CRKP repertoire carried a conserved backbone of virulence genes (*iutA*, *fyuA*, *irp2*, *fimH*, *mrk*, *iucC*, *terC*, and *traT*), indicating that carbapenemase production has not been acquired at the expense of core fitness traits. The plasmid profiles highlight the central role of mobile genetic elements in driving the evolution and persistence of CRKP in Oman and emphasise the importance of genomic surveillance to monitor the emergence and spread of high-risk plasmid-mediated resistance lineages. Cefiderocol and eravacycline remain the most promising therapeutic options against diverse CRKP carbapenemase backgrounds, whereas ceftazidime–avibactam and meropenem–vaborbactam retain important roles in the management of OXA-48-like and KPC-producing infections. The marked differences in susceptibility observed between carbapenemase groups highlight the importance of rapid carbapenemase identification to support targeted therapy and precision antimicrobial stewardship. We recommend the CZA + AZT combination for management of NDM-producing isolates, while FOS + MEM and FOS + AK may be useful for OXA-232-producing isolates. Our findings suggest that the gradient diffusion cross method may provide a simple and practical approach for assessing potentially effective antimicrobial combinations against CRKP in clinical microbiology laboratories, thereby assisting clinicians in selecting optimised combination therapy in a timely manner. The study further highlights the importance of integrating genomic resistance profiling, susceptibility testing, and phenotypic synergy assessment when evaluating treatment strategies for XDR and PDR CRKP. Such an approach is particularly relevant in settings where access to newer antimicrobial agents remains limited and optimisation of existing therapeutic options is essential.

## Figures and Tables

**Table 1:** Carbapenemases, MLST and source of infection among XDR/PDR CRKP (*n* = 38).

S. No.	Carbapenemase	MLST	Source of Infection	XDR/PDR Status
1.	OXA-232	ST-2096	Blood	XDR
2.	OXA-232	ST-231	Blood	XDR
3.	OXA-232	ST-2096	Blood	XDR
4.	OXA-232	ST-2096	Blood	XDR
5.	OXA-232	ST-2096	Suprapubic aspirate	XDR
6.	OXA-232	ST-2096	Urine	XDR
7.	OXA-232	ST-2096	Urine	XDR
8.	OXA-232	ST-2096	Urine	XDR
9.	OXA-232	ST-231	Urine	XDR
10.	OXA-232	ST-2096	Urine	XDR
11.	OXA-232	ST-2096	wound	XDR
12.	OXA-232	ST-2096	wound	XDR
13.	OXA-232	ST-2096	wound	XDR
14.	OXA-232	ST-2096	wound	XDR
15.	OXA-232	ST-2096	Pus (ear)	XDR
16.	OXA-232	ST-2096	swab	XDR
17.	OXA-232	ST-2096	swab	PDR
18.	OXA-232	ST-2096	Peritoneal fluid	XDR
19.	OXA-232	ST-2096	Tracheal aspirate	XDR
20.	OXA-232	ST-359	Tracheal aspirate	XDR
21.	OXA-232	ST-2096	Tracheal aspirate	XDR
22.	OXA-232	ST-2096	Sputum	XDR
23.	OXA-232	ST-2096	Sputum	XDR
24.	OXA-232	ST-2096	Sputum	XDR
25.	OXA-232	ST-2096	Sputum	XDR
26.	NDM-5	ST-147	Urine	PDR
27.	NDM-5	ST-147	Abdominal wound	XDR
28.	NDM-5	ST-147	wound	PDR
29.	NDM-5	ST-147	swab	XDR
30.	NDM-5	ST-147	Urine	PDR
31.	NDM-5	ST-147	Urine	XDR
32.	NDM-1 + KPC-2	ST-11	Peritoneal fluid	XDR
33.	NDM-1 + KPC-2	ST-11	Tracheal aspirate	XDR
34.	NDM-1 + KPC-2	ST-11	Blood	XDR
35.	NDM-1 + KPC-2	ST-11	Tracheal aspirate	XDR
36.	NDM-1 + KPC-2	ST-11	Tracheal aaspirate	PDR
37.	NDM-1 + KPC-2	ST-11	Blood	PDR
38.	KPC-2	ST-11	Tracheal	XDR

**Table 2:** Susceptibility profile of new antibiotics against the different CRKP.

Carbapenemase		Susceptibility No. (%)								
		New Antibiotics								
		CFD	CZA	IMR	MVP	FOS	ERV	PLZ	MIN	AZT
		Break-Point: ≤4	Break-Point: ≤8	Break-Point: ≤1	Break-Point: ≤4	Break-Point: ≤32	Break-Point: ≤0.5	Break-Point: ≤2	Break-Point: ≤4	Break-Point: ≤4
OXA-48 like	OXA232 (*n* = 25)	24 (96%)	23 (92%)	21 (84%)	10 (40%)	13 (52%)	23 (92%)	11 (44%)	12 (48%)	0
	OXA-48 like (*n* = 22)	22 (100%)	21 (96%)	21 (96%)	-	12 (54.54%)	20 (91%)	9 (40.91%)	-	-
	Total (*n* = 47)	46 (98%)	44 (94%)	42 (89.36%)	10 (40%)	25 (53.19%)	43 (92%)	20 (42.55%)	12 (48%)	0
NDM	NDM-5 (*n* = 6)	6 (100%)	0	0	0	1 (16.66%)	6 (100%)	2 (33%)	3 (50%)	0
	NDM (*n* = 22)	21 (96%)	-	-	-	0	9 (41%)	8 (36.36%)	1 (5%)	-
	Total (*n* = 28)	27 (96%)	0	0	0	1 (3.57%)	15 (54%)	10 (35.71%)	4 (14.28%)	-
KPC	KPC-2 (*n* = 1)	1 (100%)	1 (100%)	-	1 (100%)	0	1 (100%)	1 (100%)	1 (100%)	0
	KPC (*n* = 22)	19 (86%)	20 (91%)	-	20 (91%)	1	12 (55%)	8 (36.36%)	-	-
	Total (*n* = 23)	20 (87%)	21 (91%)	-	21 (91%)	1 (4.34%)	13 (57%)	9 (39.13%)	1 (100%)	0
Co- carriage	NDM-1 + KPC-2 (*n* = 6)	6 (100%)	0	3 (50%)	0	2 (33.33%)	6 (100%)	4 (66.66%)	5 (83%)	0
	NDM + KPC (*n* = 1)	1 (100%)	0	-	0	1 (100%)	1 (100%)	0	-	0
	Total (*n* = 7)	7 (100%)	0	3 (50%)	0	3 (42.85%)	7 (100%)	4 (57.14%)	5 (83%)	0
Total S %	(*n* = 105)	95.00%	62%	43%	30%	28.57%	74%	41%	37%	0

CFD: cefiderocol, CZA: ceftazidime–avibactam; IMR: imipenem relebactam; MVP: meropenem vaborbactam; FOS: fosfomycin; ERV: eravacycline, PLZ: plazomicin. MIN: minocycline, AZT: ztreonam.

**Table 3:** MIC50 and MIC90 values of newer antimicrobial agents against CRKP.

Carbapenmases	CFD		CZA		IMR		MVP		FOS		ERV		PLZ	
	Breakpoint ≤4		Breakpoint ≤8		Breakpoint ≤1		Breakpoint ≤4		Breakpoint ≤32		Breakpoint ≤0.5		Breakpoint ≤2	
	MIC50	MIC90	MIC50	MIC90	MIC50	MIC90	MIC50	MIC90	MIC50	MIC90	MIC50	MIC90	MIC50	MIC90
OXA-48 (*n* = 47)	0.125	0.5	1	2	-	-	-	-	16	≥256	0.5	0.5	64	≥256
OXA-232 (*n* = 25)	0.125	0.5	1	2	-	-	-	-	16	≥256	0.5	0.5	64	≥256
NDM (*n* = 34)	0.125	0.5	-	-	-	-	-	-	0.5	2	≥256	≥256	≥256	≥256
NDM-5 (*n* = 6)	0.064	0.5	-	-	-	-	-	-	≥256	≥256	0.5	0.5	16	≥256
KPC (*n* = 22)	0.5	1	1	4	0.5	2	0.5	2	≥256	≥256	0.25	1	≥256	≥256
KPC-2 + NDM-1 (*n* = 6)	0.5	1	≥256	≥256	1	32	32	32	≥256	≥256	0.5	0.5	2	≥256

CFD: cefiderocol; CZA: ceftazidime–avibactam; IMR: imipenem relebactam; MVP: meropenem vaborbactam; FOS: fosfomycin; ERV: eravacycline, PLZ: plazomicin.

**Table 4:** Assessment of synergistic activity of some available antimicrobials against different CRKP isolates.

Combinations	Carbapenemases	Synergy n (%)	Partial Synergy n (%)	Additive n (%)	Indifferent n (%)	Antagonism n (%)
		∑ FICI ≤ 0.50	∑ FICI = 0.5–0.75	∑ FICI = 0.76–1.0	∑ FICI = 1–4	∑ FICI > 4
CZA + AZT	OXA-232 (*n* = 25)	25 (100%)	0	0	0	0
	NDM-5 (*n* = 6)	6 (100%)	0	0	0	0
	NDM-1 + KPC-2 (*n* = 6)	6 (100%)	0	0	0	0
	KPC-2 (*n* = 1)	1 (100%)	0	0	0	0
MER + FOS	OXA-232 (*n* = 25)	8 (32%)	4 (16%)	2 (8%)	11 (44%)	0
	NDM-5 (*n* = 6)	0	1 (17%)	0	5 (83.3%)	0
	NDM-1 + KPC-2 (*n* = 6)	0	1 (17%)	0	5 (83.3%)	0
	KPC-2 (*n* = 1)	0	0	0	1 (100%)	0
AK + FOS	AK + FOS	10 (40%)	3 (12%)	1 (4%)	11 (44%)	0
	NDM-5 (*n* = 6)	0	0	0	6 (100%)	0
	NDM-1 + KPC-2 (*n* = 6)	1 (17)	0	2 (33.3%)	3 (50%)	0
	KPC-2 (*n* = 1)	0	0	0	1 (100%)	0

## Data Availability

All the data generated in this study has been provided in this paper, as either tables, figures and Supplementary Materials.

## Supplementary Materials

The following supporting information can be downloaded at: https://www.mdpi.com/article/10.3390/antibiotics15090844/s1, Document S1: E-test fixed-ratio method; Document S2: Genome Accession Numbers (Table listing BioProject, BioSample, and Whole Genome Shotgun (WGS) accession numbers for the *Klebsiella pneumoniae* isolates analyzed in this study, deposited under BioProject PRJNA1483060); Table S1: Chromosomal mutations detected among the 38 CRKP isolates; Table S2: Distribution of plasmids, MGEs, resistance, and virulence genes among XDR/PDR CRKP (*n* = 38); Table S3: Synergistic activity between CZA and AZT.

## Abbreviations

The following abbreviations used in this manuscript:

AMEs	Aminoglycoside-modifying enzymes
AZT	Aztreonam
CFD	Cefiderocol
CPE	Carbapenemase enzymes
CRE	Carbapenem-resistant Enterobacterales
CRKP	Carbapenem-resistant *Klebsiella pneumoniae*
CZA	Ceftazidime–avibactam
ERV	Eravacycline
GNB	Gram-negative bacilli
IMR	Imipenem–relebactam
KPC	*Klebsiella pneumoniae* carbapenemase
MDR	Multidrug-resistant
MGE	Mobile genetic elements
MPV	Meropenem–vaborbactam
NDM	New Delhi metallo-beta-lactamase
OXA	OXA-type carbapenemases
PLZ	Plazomicin
