# Supplementary material for: The Evolving Clonal, Plasmid-Mediated Resistome, Virulome and Therapeutic Landscape of Carbapenem-Resistant Klebsiella pneumoniae in Oman
Source: Antibiotics (Basel). 2026 Aug 31;15(9):844. doi: 10.3390/antibiotics15090844 (PMC13603273; doi:10.3390/antibiotics15090844)
Supplement: Supplementary file 1 [file antibiotics-15-00844-s001.zip › Supplementary Document S1.pdf]

Supplementary Document S1

E-test Fixed ratio method

For the combination testing procedure, E-test strips impregnated with the respective antibiotics were applied sequentially to the inoculated agar surface. Initially, E-test strip A was placed onto the bacterial lawn and allowed to incubate at ambient temperature for one hour. Following this incubation, strip A was carefully removed, and its MIC value (MICA) was recorded. Immediately afterward, E-test strip B was positioned directly over the imprint left by strip A to allow for assessment of potential synergistic effects. The plates were then incubated at 35°C for 18 hours. MIC values were interpreted by aligning the corresponding MIC scale with the gradient position of each strip, enabling precise evaluation of individual and combined drug activities

E-test fixed ratio method

In this example, minimum inhibitory concentration of A is 16 µg/ml and minimum inhibitory concentration of B is 32 µg/ml. Minimum inhibitory concentration of combination A and B is 1 µg/ml. $\sum$FIC = 0.09 (synergy). FIC: Fractional inhibitory concentration (Laishram et al., 2017). 80 3.9.3 E-test MIC: MIC method In this method, one test strip is placed on the inoculated MHA plate and incubated at room temperature for 1 h to allow diffusion of the agent. After 1 h, the agar is marked adjacent to the previously determined MIC of the agent and removed. The second E-test strip is then placed over the imprint of the previous strip such that the mark on the agar corresponds to the MIC of the second agent . The resulting ellipse of inhibition is read after 20 h of incubation at 37°C
