# Supplementary material for: The Evolving Clonal, Plasmid-Mediated Resistome, Virulome and Therapeutic Landscape of Carbapenem-Resistant Klebsiella pneumoniae in Oman
Source: Antibiotics (Basel). 2026 Aug 31;15(9):844. doi: 10.3390/antibiotics15090844 (PMC13603273; doi:10.3390/antibiotics15090844)
Supplement: Supplementary file 1 [file antibiotics-15-00844-s001.zip › Supplementary Document S2.pdf]

Supplementary Document S2
Genome Accession Numbers

The table below lists BioProject, BioSample, and Whole Genome Shotgun (WGS) accession numbers for the *Klebsiella pneumoniae* isolates analyzed in this study, deposited under BioProject PRJNA1483060.

SUBID	BioProject	BioSample	Accession	Organism
SUB16281942	PRJNA1483060	SAMN61142006	JCAFKC000000000	Klebsiella pneumoniae 40
SUB16281942	PRJNA1483060	SAMN61142005	JCAFKD000000000	Klebsiella pneumoniae 39
SUB16281942	PRJNA1483060	SAMN61142004	JCAFKE000000000	Klebsiella pneumoniae 38
SUB16281942	PRJNA1483060	SAMN61142003	JCAFKF000000000	Klebsiella pneumoniae 37
SUB16281942	PRJNA1483060	SAMN61142002	JCAFKG000000000	Klebsiella pneumoniae 36
SUB16281942	PRJNA1483060	SAMN61142001	JCAFKH000000000	Klebsiella pneumoniae 35
SUB16281942	PRJNA1483060	SAMN61142000	JCAFKI000000000	Klebsiella pneumoniae 34
SUB16281942	PRJNA1483060	SAMN61141999	JCAFKJ000000000	Klebsiella pneumoniae 33
SUB16281942	PRJNA1483060	SAMN61141998	JCAFKK000000000	Klebsiella pneumoniae 32
SUB16281942	PRJNA1483060	SAMN61141997	JCAFKL000000000	Klebsiella pneumoniae 31
SUB16281942	PRJNA1483060	SAMN61141996	JCAFKM000000000	Klebsiella pneumoniae 30
SUB16281942	PRJNA1483060	SAMN61141995	JCAFKN000000000	Klebsiella pneumoniae 29
SUB16281942	PRJNA1483060	SAMN61141994	JCAFKO000000000	Klebsiella pneumoniae 28
SUB16281942	PRJNA1483060	SAMN61141993	JCAFKP000000000	Klebsiella pneumoniae 27
SUB16281942	PRJNA1483060	SAMN61141992	JCAFKQ000000000	Klebsiella pneumoniae 26
SUB16281942	PRJNA1483060	SAMN61141991	JCAFKR000000000	Klebsiella pneumoniae 25
SUB16281942	PRJNA1483060	SAMN61141990	JCAFKS000000000	Klebsiella pneumoniae 24
SUB16281942	PRJNA1483060	SAMN61141989	JCAFKT000000000	Klebsiella pneumoniae 23
SUB16281942	PRJNA1483060	SAMN61141988	JCAFKU000000000	Klebsiella pneumoniae 22
SUB16281942	PRJNA1483060	SAMN61141987	JCAFKV000000000	Klebsiella pneumoniae 21
SUB16281942	PRJNA1483060	SAMN61141986	JCAFKW000000000	Klebsiella pneumoniae 20
SUB16281942	PRJNA1483060	SAMN61141985	JCAFKX000000000	Klebsiella pneumoniae 19
SUB16281942	PRJNA1483060	SAMN61141984	JCAFKY000000000	Klebsiella pneumoniae 18
SUB16281942	PRJNA1483060	SAMN61141983	JCAFKZ000000000	Klebsiella pneumoniae 17
SUB16281942	PRJNA1483060	SAMN61141982	JCAFLA000000000	Klebsiella pneumoniae 16
SUB16281942	PRJNA1483060	SAMN61141981	JCAFLB000000000	Klebsiella pneumoniae 15
SUB16281942	PRJNA1483060	SAMN61141980	JCAFLC000000000	Klebsiella pneumoniae 14
SUB16281942	PRJNA1483060	SAMN61141979	JCAFLD000000000	Klebsiella pneumoniae 13
SUB16281942	PRJNA1483060	SAMN61141978	JCAFLE000000000	Klebsiella pneumoniae 12
SUB16281942	PRJNA1483060	SAMN61141977	JCAFLF000000000	Klebsiella pneumoniae 11
