# Supplementary material for: The Evolving Clonal, Plasmid-Mediated Resistome, Virulome and Therapeutic Landscape of Carbapenem-Resistant Klebsiella pneumoniae in Oman
Source: Antibiotics (Basel). 2026 Aug 31;15(9):844. doi: 10.3390/antibiotics15090844 (PMC13603273; doi:10.3390/antibiotics15090844)
Supplement: Supplementary file 1 [file antibiotics-15-00844-s001.zip › Supplementary Table S1.pdf]

Supplementary Table S1: Chromosomal mutations detected among the 38 CRKP isolates.

Isolate	Gene	Mutation	Antibiotic
**K.P1**	*acrR*	[p.P161R, p.G164A, p.F172S, p.R173G, p.L195V, p.F197I, p.K201M]	Fluoroquinolone
	gyrA	[p.D87G]	Fluoroquinolone
	parC	[p.S80I]	Fluoroquinolone
	ompK36	[p.N49S, p.L59V, p.A190W - Frameshift, p.L191S, p.F207W, p.D224E, p.Q227S - Frameshift, p.L228V, p.E232R, p.T254S, p.N304E - Frameshift]	Cephalosporins
	ompK36	[p.A217S, p.N218H]	Carbapenem
	ompK37	[p.I70M, p.I128M]	Carbapenem
**K.P2**	*acrR*	[p.P161R, p.G164A, p.F172S, p.R173G, p.L195V, p.F197I, p.K201M]	Fluoroquinolone
	parC	[p.S80I]	Fluoroquinolone
	ompK36	[p.N49S, p.L59V, p.T184P]	Cephalosporins
	ompK37	[p.I70M, p.I128M, p.N230G, p.M233Q - Frameshift]	Carbapenem
**K.P3**	*acrR*	[p.P161R, p.G164A, p.F172S, p.R173G, p.L195V, p.F197I, p.K201M]	Fluoroquinolone
	parC	[p.S80I]	Fluoroquinolone
	ompK36	[p.N49S, p.L59V, p.G189T, p.F198Y, p.F207Y, p.T222L, p.D223G, p.Q227S - Frameshift, p.L228K - Frame restored, p.E232R, p.N304E]	Cephalosporins
	ompK36	[p.A217S]	Carbapenem
	ompK37	[p.I70M, p.I128M, p.N230G, p.M233Q - Frameshift]	Carbapenem
**K.P4**	*acrR*	[p.P161R, p.G164A, p.F172S, p.R173G, p.L195V, p.F197I, p.K201M]	Fluoroquinolone
	gyrA	[p.D87G]	Fluoroquinolone
	parC	[p.S80I]	Fluoroquinolone
	ompK36	[p.N49S, p.L59V, p.A190W - Frameshift, p.L191S, p.F207W, p.D224E, p.Q227S - Frameshift, p.L228V, p.E232R, p.T254S, p.N304E - Frameshift]	Cephalosporins
	ompK36	[p.A217S, p.N218H]	Carbapenem
	ompK37	[p.I70M, p.I128M]	Carbapenem

Isolate	Gene	Mutation	Antibiotic
**K.P5**	*acrR*	[p.P161R, p.G164A, p.F172S, p.R173G, p.L195V, p.F197I, p.K201M]	Fluoroquinolone
	gyrA	[p.D87G]	Fluoroquinolone
	parC	[p.S80I]	Fluoroquinolone
	ompK36	[p.N49S, p.L59V, p.T184P]	Cephalosporins
	ompK37	[p.I70M, p.I128M, p.N230G, p.M233Q - Frameshift]	Carbapenem
**K.P6**	*acrR*	[p.P161R, p.G164A, p.F172S, p.R173G, p.L195V, p.F197I, p.K201M]	Fluoroquinolone
	gyrA	[p.D87G]	Fluoroquinolone
	parC	[p.S80I]	Fluoroquinolone
	ompK36	[p.N49S, p.L59V, p.A190W - Frameshift, p.L191S, p.F207W, p.D224E, p.Q227S - Frameshift, p.L228V, p.E232R, p.T254S, p.N304E - Frameshift]	Cephalosporins
	ompK36	[p.A217S, p.N218H]	Carbapenem
	ompK37	[p.I70M, p.I128M]	Carbapenem
**K.P7**	*acrR*	[p.P161R, p.G164A, p.F172S, p.R173G, p.L195V, p.F197I, p.K201M]	Fluoroquinolone
	gyrA	[p.D87G]	Fluoroquinolone
	parC	[p.S80I]	Fluoroquinolone
	ompK36	[p.N49S, p.L59V, p.A190W - Frameshift, p.L191S, p.F207W, p.D224E, p.Q227S - Frameshift, p.L228V, p.E232R, p.T254S, p.N304E - Frameshift]	Cephalosporins
	ompK36	[p.A217S, p.N218H]	Carbapenem
	ompK37	[p.I70M, p.I128M]	Carbapenem
**K.P8**	*acrR*	[p.P161R, p.G164A, p.F172S, p.R173G, p.L195V, p.F197I, p.K201M]	Fluoroquinolone
	gyrA	[p.D87G]	Fluoroquinolone
	parC	[p.S80I]	Fluoroquinolone
	ompK36	[p.N49S, p.L59V, p.A190W - Frameshift, p.L191S, p.F207W, p.D224E, p.Q227S - Frameshift, p.L228V, p.E232R, p.T254S, p.N304E - Frameshift]	Cephalosporins
	ompK36	[p.A217S, p.N218H]	Carbapenem
	ompK37	[p.I70M, p.I128M]	Carbapenem

Isolate	Gene	Mutation	Antibiotic
**K.P9**	*acrR*	[p.P161R, p.G164A, p.F172S, p.R173G, p.L195V, p.F197I, p.K201M]	Fluoroquinolone
	gyrA	[p.D87G]	Fluoroquinolone
	parC	[p.S80I]	Fluoroquinolone
	ompK36	[p.N49S, p.L59V, p.A190W - Frameshift, p.L191S, p.F207W, p.D224E, p.Q227S - Frameshift, p.L228V, p.E232R, p.T254S, p.N304E - Frameshift]	Cephalosporins
	ompK36	[p.A217S, p.N218H]	Carbapenem
	ompK37	[p.I70M, p.I128M]	Carbapenem
**K.P10**	*acrR*	[p.P161R, p.G164A, p.F172S, p.R173G, p.L195V, p.F197I, p.K201M]	Fluoroquinolone
	gyrA	[p.D87G]	Fluoroquinolone
	parC	[p.S80I]	Fluoroquinolone
	ompK36	[p.N49S, p.L59V, p.A190W - Frameshift, p.L191S, p.F207W, p.D224E, p.Q227S - Frameshift, p.L228V, p.E232R, p.T254S, p.N304E - Frameshift]	Cephalosporins
	ompK36	[p.A217S, p.N218H]	Carbapenem
	ompK37	[p.I70M, p.I128M]	Carbapenem
**K.P12**	*acrR*	[p.P161R, p.G164A, p.F172S, p.R173G, p.L195V, p.F197I, p.K201M]	Fluoroquinolone
	gyrA	[p.D87G]	Fluoroquinolone
	parC	[p.S80I]	Fluoroquinolone
	ompK36	[p.N49S, p.L59V, p.A190W - Frameshift, p.L191S, p.F207W, p.D224E, p.Q227S - Frameshift, p.L228V, p.E232R, p.T254S, p.N304E - Frameshift]	Cephalosporins
	ompK36	[p.A217S, p.N218H]	Carbapenem
	ompK37	[p.I70M, p.I128M]	Carbapenem
**K.P13**	*acrR*	[p.P161R, p.G164A, p.F172S, p.R173G, p.L195V, p.F197I, p.K201M]	Fluoroquinolone
	gyrA	[p.D87G]	Fluoroquinolone
	parC	[p.S80I]	Fluoroquinolone
	ompK36	[p.N49S, p.L59V, p.A190W - Frameshift, p.L191S, p.F207W, p.D224E, p.Q227S - Frameshift, p.L228V, p.E232R, p.T254S, p.N304E - Frameshift]	Cephalosporins

Isolate	Gene	Mutation	Antibiotic
	ompK36	[p.A217S, p.N218H]	Carbapenem
	ompK37	[p.I70M, p.I128M]	Carbapenem
**K.P14**	*acrR*	[p.P161R, p.G164A, p.F172S, p.R173G, p.L195V, p.F197I, p.K201M]	Fluoroquinolone
	gyrA	[p.D87G]	Fluoroquinolone
	parC	[p.S80I]	Fluoroquinolone
	ompK36	[p.N49S, p.L59V, p.A190W - Frameshift, p.L191S, p.F207W, p.D224E, p.Q227S - Frameshift, p.L228V, p.E232R, p.T254S, p.N304E - Frameshift]	Cephalosporins
	ompK36	[p.A217S, p.N218H]	Carbapenem
	ompK37	[p.I70M, p.I128M]	Carbapenem
**K.P15**	*acrR*	[p.P161R, p.G164A, p.F172S, p.R173G, p.L195V, p.F197I, p.K201M]	Fluoroquinolone
	parC	[p.S80I]	Fluoroquinolone
	ompK36	[p.N49S, p.L59V, p.G189T, p.F198Y, p.F207Y, p.T222L, p.D223G, p.Q227S - Frameshift, p.L228K - Frame restored, p.E232R, p.N304E]	Cephalosporins
	ompK36	[p.A217S]	Carbapenem
	ompK37	[p.I70M, p.I128M]	Carbapenem
**K.P16**	*acrR*	[p.P161R, p.G164A, p.F172S, p.R173G, p.L195V, p.F197I, p.K201M]	Fluoroquinolone
	gyrA	[p.D87G]	Fluoroquinolone
	parC	[p.S80I]	Fluoroquinolone
	ompK36	[p.N49S, p.L59V, p.A190W - Frameshift, p.L191S, p.F207W, p.D224E, p.Q227S - Frameshift, p.L228V, p.E232R, p.T254S, p.N304E - Frameshift]	Cephalosporins
	ompK36	[p.A217S, p.N218H]	Carbapenem
	ompK37	[p.I70M, p.I128M]	Carbapenem
**K.P17**	*acrR*	[p.P161R, p.G164A, p.F172S, p.R173G, p.L195V, p.F197I, p.K201M]	Fluoroquinolone
	gyrA	[p.D87G]	Fluoroquinolone
	parC	[p.S80I]	Fluoroquinolone

Isolate	Gene	Mutation	Antibiotic
	ompK36	[p.N49S, p.L59V, p.A190W - Frameshift, p.L191S, p.F207W, p.D224E, p.Q227S - Frameshift, p.L228V, p.E232R, p.T254S, p.N304E - Frameshift]	Cephalosporins
	ompK36	[p.A217S, p.N218H]	Carbapenem
	ompK37	[p.I70M, p.I128M]	Carbapenem
**K.P18**	*acrR*	[p.P161R, p.G164A, p.F172S, p.R173G, p.L195V, p.F197I, p.K201M]	Fluoroquinolone
	parC	[p.S80I]	Fluoroquinolone
	ompK36	[p.N49S, p.L59V, p.T184P]	Cephalosporins
	ompK37	[p.I70M, p.I128M, p.N230G, p.M233Q - Frameshift]	Carbapenem
**K.P19**	*acrR*	[p.P161R, p.G164A, p.F172S, p.R173G, p.L195V, p.F197I, p.K201M]	Fluoroquinolone
	gyrA	[p.D87G]	Fluoroquinolone
	parC	[p.S80I]	Fluoroquinolone
	ompK36	[p.N49S, p.L59V, p.A190W - Frameshift, p.L191S, p.F207W, p.D224E, p.Q227S - Frameshift, p.L228V, p.E232R, p.T254S, p.N304E - Frameshift]	Cephalosporins
	ompK36	[p.A217S, p.N218H]	Carbapenem
	ompK37	[p.I70M, p.I128M]	Carbapenem
**K.P20**	*acrR*	[p.P161R, p.G164A, p.F172S, p.R173G, p.L195V, p.F197I, p.K201M]	Fluoroquinolone
	gyrA	[p.D87G]	Fluoroquinolone
	parC	[p.S80I]	Fluoroquinolone
	ompK36	[p.N49S, p.L59V, p.A190W - Frameshift, p.L191S, p.F207W, p.D224E, p.Q227S - Frameshift, p.L228V, p.E232R, p.T254S, p.N304E - Frameshift]	Cephalosporins
	ompK36	[p.A217S, p.N218H]	Carbapenem
	ompK37	[p.I70M, p.I128M]	Carbapenem
**K.P21**	*acrR*	[p.P161R, p.G164A, p.F172S, p.R173G, p.L195V, p.F197I, p.K201M]	Fluoroquinolone
	gyrA	[p.D87G],	Fluoroquinolone
	parC	[p.S80I]	Fluoroquinolone

Isolate	Gene	Mutation	Antibiotic
	ompK36	[p.N49S, p.L59V, p.A190W - Frameshift, p.L191S, p.F207W, p.D224E, p.Q227S - Frameshift, p.L228V, p.E232R, p.T254S, p.N304E - Frameshift]	Cephalosporins
	ompK36	[p.A217S, p.N218H]	Carbapenem
	ompK37	[p.I70M, p.I128M]	Carbapenem
**K.P22**	*acrR*	[p.P161R, p.G164A, p.F172S, p.R173G, p.L195V, p.F197I, p.K201M]	Fluoroquinolone
	gyrA	[p.D87G],	Fluoroquinolone
	parC	[p.S80I]	Fluoroquinolone
	ompK36	[p.N49S, p.L59V, p.T184P]	Cephalosporins
	ompK37	[p.I70M, p.I128M, p.N230G, p.M233Q - Frameshift]	Carbapenem
**K.P23**	*acrR*	[p.P161R, p.G164A, p.F172S, p.R173G, p.L195V, p.F197I, p.K201M]	Fluoroquinolone
	parC	[p.S80I]	Fluoroquinolone
	ompK36	[p.N49S, p.L59V, p.G189T, p.F198Y, p.F207Y, p.T222L, p.D223G, p.Q227S - Frameshift, p.L228K - Frame restored, p.E232R, p.N304E]	Cephalosporins
	ompK36	[p.A217S]	Carbapenem
	ompK37	[p.I70M, p.I128M]	Carbapenem
**K.P24**	*acrR*	[p.P161R, p.G164A, p.F172S, p.R173G, p.L195V, p.F197I, p.K201M]	Fluoroquinolone
	parC	[p.S80I]	Fluoroquinolone
	ompK36	[p.N49S, p.L59V, p.G189T, p.F198Y, p.F207Y, p.T222L, p.D223G, p.Q227S - Frameshift, p.L228K - Frame restored, p.E232R, p.N304E]	Cephalosporins
	ompK36	[p.A217S]	Carbapenem
	ompK37	[p.I70M, p.I128M]	Carbapenem
**K.P25**	*acrR*	[p.P161R, p.G164A, p.F172S, p.R173G, p.L195V, p.F197I, p.K201M]	Fluoroquinolone
	gyrA	[p.D87G],	Fluoroquinolone
	parC	[p.S80I]	Fluoroquinolone
	ompK36	[p.N49S, p.L59V, p.T184P]	Cephalosporins
**K.P26**	*acrR*	[p.P161R, p.G164A, p.F172S, p.R173G, p.L195V, p.F197I, p.K201M]	Fluoroquinolone

Isolate	Gene	Mutation	Antibiotic
	gyrA	[p.D87G],	Fluoroquinolone
	parC	[p.S80I]	Fluoroquinolone
	ompK36	[p.N49S, p.L59V, p.T184P]	Cephalosporins
	ompK37	[p.I70M, p.I128M, p.N230G, p.M233Q - Frameshift]	Carbapenem
**K.P27**	*gyrA*	[p.D87G],	Fluoroquinolone
	parC	[p.S80I]	Fluoroquinolone
	ompK36	[p.N49S, p.L59V, p.A190W - Frameshift, p.L191S, p.F207W, p.D224E, p.Q227S - Frameshift, p.L228V, p.E232R, p.T254S, p.N304E - Frameshift]	Cephalosporins
	ompK36	[p.A217S, p.N218H],	Carbapenem
	ompK37	[p.I70M, p.I128M]	Carbapenem
**K.P28**	*acrR*	[p.P161R, p.G164A, p.F172S, p.R173G, p.L195V, p.F197I, p.K201M]	Fluoroquinolone
	gyrA	[p.D87G],	Fluoroquinolone
	parC	[p.S80I]	Fluoroquinolone
	ompK36	[p.N49S, p.L59V, p.T184P]	Cephalosporins
	ompK37	[p.I70M, p.I128M, p.N230G, p.M233Q - Frameshift]	Carbapenem
**K.P29**	*acrR*	[p.P161R, p.G164A, p.F172S, p.R173G, p.L195V, p.F197I, p.K201M]	Fluoroquinolone
	gyrA	[p.D87G],	Fluoroquinolone
	parC	[p.S80I]	Fluoroquinolone
	ompK36	[p.N49S, p.L59V, p.T184P]	Cephalosporins
	ompK37	[p.I70M, p.I128M, p.N230G, p.M233Q - Frameshift]	Carbapenem
**K.P30**	*acrR*	[p.P161R, p.G164A, p.F172S, p.R173G, p.L195V, p.F197I, p.K201M]	Fluoroquinolone
	gyrA	[p.D87G],	Fluoroquinolone
	parC	[p.S80I]	Fluoroquinolone
	ompK36	[p.N49S, p.L59V, p.T184P]	Cephalosporins
	ompK37	[p.I70M, p.I128M, p.N230G, p.M233Q - Frameshift]	Carbapenem
**K.P31**	*acrR*	p.P161R, p.G164A, p.F172S, p.R173G, p.L195V, p.F197I, p.K201M]	Fluoroquinolone

Isolate	Gene	Mutation	Antibiotic
	gyrA	[p.D87G],	Fluoroquinolone
	parC	[p.S80I]	Fluoroquinolone
	ompK36	[p.N49S, p.L59V, p.A190W - Frameshift, p.L191S, p.F207W, p.D224E, p.Q227S - Frameshift, p.L228V, p.E232R, p.T254S, p.N304E - Frameshift]	Cephalosporins
	ompK36	[p.A217S, p.N218H],	Carbapenem
	ompK37	[p.I70M, p.I128M]	Carbapenem
**K.P32**	*acrR*	[p.P161R, p.G164A, p.F172S, p.R173G, p.L195V, p.F197I, p.K201M]	Fluoroquinolone
	gyrA	[p.D87G],	Fluoroquinolone
	parC	[p.S80I]	Fluoroquinolone
	ompK36	[p.N49S, p.L59V, p.A190W - Frameshift, p.L191S, p.F207W, p.D224E, p.Q227S - Frameshift, p.L228V, p.E232R, p.T254S, p.N304E - Frameshift]	Cephalosporins
	ompK36	[p.A217S, p.N218H],	Carbapenem
	ompK37	[p.I70M, p.I128M]	Carbapenem
**K.P33**	*acrR*	[p.P161R, p.G164A, p.F172S, p.R173G, p.L195V, p.F197I, p.K201M]	Fluoroquinolone
	gyrA	[p.D87G],	Fluoroquinolone
	parC	[p.S80I]	Fluoroquinolone
	ompK36	[p.A217S, p.N218H],	Carbapenem
	ompK37	[p.I70M, p.I128M]	Carbapenem
**K.P35**	*acrR*	[p.P161R, p.G164A, p.F172S, p.R173G, p.L195V, p.F197I, p.K201M]	Fluoroquinolone
	gyrA	[p.D87G],	Fluoroquinolone
	parC	[p.S80I]	Fluoroquinolone
	ompK36	[p.N49S, p.L59V, p.A190W - Frameshift, p.L191S, p.F207W, p.D224E, p.Q227S - Frameshift, p.L228V, p.E232R, p.T254S, p.N304E - Frameshift]	Cephalosporins
	ompK36	[p.A217S, p.N218H],	Carbapenem
	ompK37	[p.I70M, p.I128M]	Carbapenem
**K.P36**	*acrR*	[p.P161R, p.G164A, p.F172S, p.R173G, p.L195V, p.F197I, p.K201M]	Fluoroquinolone

Isolate	Gene	Mutation	Antibiotic
	parC	[p.S80I]	Fluoroquinolone
	ompK36	[p.N49S, p.L59V, p.G189T, p.F198Y, p.F207Y, p.T222L, p.D223G, p.Q227S - Frameshift, p.L228K - Frame restored, p.E232R, p.N304E]	Cephalosporins
	ompK36	[p.A217S],	Carbapenem
	ompK37	[p.I70M, p.I128M]	Carbapenem
**K.P37**	*acrR*	[p.P161R, p.G164A, p.F172S, p.R173G, p.L195V, p.F197I, p.K201M]	Fluoroquinolone
	gyrA	[p.D87G],	Fluoroquinolone
	parC	[p.S80I]	Fluoroquinolone
	ompK36	[p.N49S, p.L59V, p.A190W - Frameshift, p.L191S, p.F207W, p.D224E, p.Q227S - Frameshift, p.L228V, p.E232R, p.T254S, p.N304E - Frameshift]	Cephalosporins
	ompK36	[p.A217S, p.N218H],	Carbapenem
	ompK37	[p.I70M, p.I128M]	Carbapenem
**K.P38**	*acrR*	[p.P161R, p.G164A, p.F172S, p.R173G, p.L195V, p.F197I, p.K201M]	Fluoroquinolone
	parC	[p.S80I]	Fluoroquinolone
	ompK36	[p.N49S, p.L59V, p.G189T, p.F198Y, p.F207Y, p.T222L, p.D223G, p.Q227S - Frameshift, p.L228K - Frame restored, p.E232R, p.N304E]	Cephalosporins
	ompK36	[p.A217S],	Carbapenem
	ompK37	[p.I70M, p.I128M]	Carbapenem
**K.P39**	*acrR*	[p.P161R, p.G164A, p.F172S, p.R173G, p.L195V, p.F197I, p.K201M]	Fluoroquinolone
	gyrA	[p.D87G],	Fluoroquinolone
	parC	[p.S80I]	Fluoroquinolone
	ompK36	[p.N49S, p.L59V, p.A190W - Frameshift, p.L191S, p.F207W, p.D224E, p.Q227S - Frameshift, p.L228V, p.E232R, p.T254S, p.N304E - Frameshift]	Cephalosporins
	ompK36	[p.A217S, p.N218H],	Carbapenem
	ompK37	[p.I70M, p.I128M]	Carbapenem
**K.P40**	*acrR*	[p.P161R, p.G164A, p.F172S, p.R173G, p.L195V, p.F197I, p.K201M]	Fluoroquinolone

Isolate	Gene	Mutation	Antibiotic
	parC	[p.S80I]	Fluoroquinolone
	ompK36	[p.N49S, p.L59V, p.G189T, p.F198Y, p.F207Y, p.T222L, p.D223G, p.Q227S - Frameshift, p.L228K - Frame restored, p.E232R, p.N304E]	Cephalosporins
	ompK36	[p.A217S],	Carbapenem
	ompK37	[p.I70M, p.I128M]	Carbapenem
