# Supplementary material for: The Evolving Clonal, Plasmid-Mediated Resistome, Virulome and Therapeutic Landscape of Carbapenem-Resistant Klebsiella pneumoniae in Oman
Source: Antibiotics (Basel). 2026 Aug 31;15(9):844. doi: 10.3390/antibiotics15090844 (PMC13603273; doi:10.3390/antibiotics15090844)
Supplement: Supplementary file 1 [file antibiotics-15-00844-s001.zip › Supplementary Table S2.pdf]

Supplementary Table S2. Distribution of Plasmids, MGEs, Resistance and virulence genes among XDR/PDR CRKP (n=38).

CRKP	Plasmid	MGE	Resistance genes	Virulence genes
K.p1	ColKP3, IncFIB(Mar),IncHI1B, IncFIB(K), ColRNAI, Col440I	16	***blaOXA-232,****blaSHV-28, blaSHV-106, catB3, catB3, msr(E),* ***armA****, dfrA1, OqxB, OqxA, blaTEM-1A, tet(D), fosA, dfrA14,blaCTX-M-15*	*traT, iucC, terC, fyuA, irp2, mrkA:ABW83989, fimH, iutA*
K.p2	ColKP3,IncFIA,Col440I, IncFIB(pQil), ColRNAI, IncFII(K), IncFII(pAMA1167-NDM-5)	13	***blaOXA-232****,fosA, blaTEM-1B, qnrS, erm(B), ARR-2, OqxB, aac(6')-Ib-cr, aac(6')-Ib-Hangzhou,****rmtF,*** *mph(A), qacE,sul1,catA1, aadA2, dfrA12,OqxA, blaSHV-28, blaSHV-106, blaCTX-M-15, sitABCD*	*sitA, iucC, iutA, traT, fimH, mrkA:ABW83989, fyuA, irp2, nlpI*
K.p3	ColKP3, IncHI1B, Col440II, Col440I, IncFIB(Mar), IncFIB(pQil)	10	***blaOXA-232****,fosA, OqxB, OqxA, catB3, blaOXA-1,aac(6')-Ib-cr, catB3, aac(3)-Iid, catA1, blaSHV-182, blaCTX-M-15,*	*fimH, mrkA:ABW83989, fyuA, irp2, iutA, terC, nlpI*
K.p4	ColKP3, IncFIB(K), IncFIB(Mar), Col440I, IncHI1B, ColRNAI	17	***blaOXA-232,*** *blaSHV-28, blaSHV-106,catB3, blaOXA-1, aac(6')-Ib-cr, msr(E),* ***armA,*** *sul1, dfrA12, aadA2, qacE, mphE, dfrA1, OqxB, OqxA, blaTEM-1A, tet(D), fosA,dfrA14, blaCTX-M-15*	*mrkA:ABW83989, terC, iucC, iutA, traT, fyuA, irp2, fimH*
K.p5	ColRNAI, IncHI1B, IncR, IncFIB(Mar), IncFII(pHN7A8), ColRNAI	18	*sul2, fosA, OqxB, OqxA,* ***blaKPC-2****, aph(3')-VI, qnrS1, blaSHV-12, blaCTX-M-65, fosA3,* ***armA****, mphE, msr*	*fimH, mrkA:ABW83989, iucC, iutA, fyuA, irp2, ccI, traJ*
K.p6	ColKP3, IncHI1B, ColRNAI, Col440I, IncFIB(Mar)	21	***blaOXA-232,****OqxB, OqxA, blaSHV-28, blaSHV-106, blaCTX-M-15, blaOXA-1, fosA, dfrA1,dfrA12,* ***armA****, msr(E),qacE,mphE,sul1, aadA2 , catB3, aac(6')-Ib-cr*	*mrkA:ABW83989, iutA, fimH, terC, fyuA, irp2, traT, iucC*
K.p7	ColKP3, IncFIB(K), IncHI1B, ColRNAI, Col440I, IncFIB(Mar)	19	***blaOXA-232,*** *blaSHV-28, blaSHV-106, catB3, blaOXA-1, aac(6')-Ib-cr, msrE,* ***armA,****sul1, dfrA12, aadA2, qacE, mphE, dfrA1, OqxB, OqxA, blaTEM-1A, tet(D), fosA, dfrA14, blaCTX-M-15*	*mrkA:ABW83989, iutA, fimH, terC, fyuA, irp2, traT, iucC*

K.p8	ColKP3, Col440I, ColRNAI, IncFIB(Mar), IncHI1B, IncFIB(K)	18	***blaOXA-232***,*blaSHV-28, blaSHV-106, catB3, blaOXA-1, aac(6')-Ib-cr, msrE,* ***armA,****sul1, dfrA12, aadA2, qacE, mphE, dfrA1, OqxB, OqxA, blaTEM-1A, tet(D), fosA, dfrA14, blaCTX-M-15*	*mrkA:ABW83989, iutA, fimH, terC, fyuA, irp2, traT, iucC*
K.p9	ColKP3, IncFIB(K), IncFIB(Mar), Col440I, IncHI1B, ColRNAI	22	***blaOXA-232,****blaSHV-28, blaSHV-106, catB3, blaOXA-1, aac(6')-Ib-cr, msrE,* ***armA,****sul1, dfrA12, aadA2, qacE, mphE, dfrA1, OqxB, OqxA, blaTEM-1A, tet(D), fosA, dfrA14, blaCTX-M-16*	*mrkA:ABW83989, iutA, fimH, terC, fyuA, irp2, traT, iucC*
K.p10	ColKP3, IncFIB(K), Col440I, IncFIB(Mar), ColRNAI, IncHI1B	20	***blaOXA-232,*** *blaSHV-28, blaSHV-106, catB3, blaOXA-1, aac(6')-Ib-cr, msrE,* ***armA****,sul1, dfrA12, aadA2, qacE, mphE, dfrA1, OqxB, OqxA, blaTEM-1A, tet(D), fosA, dfrA14, blaCTX-M-17*	*mrkA:ABW83989, iutA, fimH, terC, fyuA, irp2, traT, iucC, colE8, colE2-like*
K.p12	ColKP3, IncFIB(K), Col440I, ColRNAI	15	***blaOXA-232,****blaOXA-1, blaSHV-28, blaSHV-106, blaCTX-M-15, fosA,OqxB, OqxA, dfrA1, catB3,aac(6')-Ib-cr*	*mrkA:ABW83989, iutA, fimH, fyuA, irp2, traT*
K.p13	ColKP3, IncFIB(K), ColRNAI, IncFIB(Mar),Col440I, IncHI1B	19	***blaOXA-232,****OqxB, OqxA, blaSHV-28, blaSHV-106, blaCTX-M-15, blaOXA-1, fosA, dfrA1,dfrA12,* ***armA,*** *msr(E), qacE,mphE,sul1,aadA2 , catB3, aac(6')-Ib-cr*	*mrkA:ABW83989, iutA, fimH, terC, fyuA, irp2, traT, iucC*
K.p14	ColKP3, IncFIB(K), IncHI1B, ColRNAI, Col440I, IncFIB(Mar)	23	***blaOXA-232,****OqxB, OqxA, blaSHV-28, blaSHV-106, blaCTX-M-15, blaOXA-1, fosA, dfrA1,dfrA12,* ***armA,*** *msr(E), qacE,mphE,sul1,aadA2 , catB3, aac(6')-Ib-cr*	*mrkA:ABW83989, iutA, fimH, terC, fyuA, irp2, traT, iucC*
K.p15	IncHI1B, IncFIB(K), Col440I, IncFIB(pKPHS1), IncR, IncFIB(Mar)	29	***blaNDM-5,*** *blaTEM-1B, blaOXA-9, aadA1, aac(6')-Ib, blaTEM-1C, aac(6')-Ib-cr, OqxB, OqxA, catA1, msr€, aph(3')-VI,* ***armA,*** *qnrS1, mphA, mph(A), dfrA5, sul1, qacE, blaSHV-67, blaSHV-11, sul2, aph(3')-Ia, fosA, blaCTX-M-15*	*terC, iutA, fimH, nlpI,iucC, iutA, irp2, fyuA, clpK1, mrkA:ABW83989*

K.p16	ColKP3, IncFIB(K), IncFIB(Mar), Col440I, IncHI1B, ColRNAI	21	***blaOXA-232,*** *OqxB, OqxA, blaSHV-28, blaSHV-106, blaCTX-M-15, blaOXA-1, fosA, dfrA1,dfrA12,* ***armA****, msr(E), qacE,mphE,sul1,aadA2 , catB3, aac(6')-Ib-cr*	*mrkA:ABW83989, iutA, fimH, terC, fyuA, irp2, traT, iucC*
K.p17	ColKP3, IncFIB(Mar), IncHI1B, Col440I, ColRNAI, IncFIB(K)	20	***blaOXA-232,*** *blaSHV-28, blaSHV-106, catB3, blaOXA-1, aac(6')-Ib-cr, msr€,* ***armA****,sul1, dfrA12, aadA2, qacE, mphE, dfrA1, OqxB, OqxA, blaTEM-1A, tet(D), fosA, dfrA14, blaCTX-M-15*	*mrkA:ABW83989, iutA, fimH, terC, fyuA, irp2, traT, iucC*
K.p18	IncFII(K), ColKP3, Col440I, IncX4, IncFIB(pQil), IncFIB(K)	24	***blaOXA-232,****blaTEM-1B, aadA1,* ***rmtF,*** *aac(6')-Ib-Hangzhou, ARR-2, aac(6')-Ib-cr, tet(A),qacE, sul1, dfrA5, qnrA1, cmlA1, catA1, blaSHV-75, blaSHV-67, blaSHV-11, mph(A), OqxB, aph(3')-Ia, OqxA, fosA, blaCTX-M-15*	*fimH, mrkA:ABW83989, nlpI, traT, irp2, fyuA, iutA*
K.p19	ColKP3, IncHI1B, ColRNAI, IncFIB(K), Col440I, IncFIB(Mar)	20	***blaOXA-232,*** *blaSHV-28, blaSHV-106, catB3, blaOXA-1, aac(6')-Ib-cr, , msr€,* ***armA****,sul1, dfrA12, aadA2, qacE, mphE, dfrA1, OqxB, OqxA, blaTEM-1A, tet(D), fosA, dfrA14, blaCTX-M-15*	*mrkA:ABW83989, iutA, fimH, terC, fyuA, irp2, traT, iucC*
K.p20	ColKP3, IncFIB(K), IncFIB(Mar), ColRNAI, Col440I, IncHI1B	20	***blaOXA-232,*** *blaSHV-28, blaSHV-106, catB3, blaOXA-1, aac(6')-Ib-cr, msrE,* ***armA****,sul1, dfrA12, aadA2, qacE, mphE, dfrA1, OqxB, OqxA, blaTEM-1A, tet(D), fosA, dfrA14, blaCTX-M-15*	*mrkA:ABW83989, iutA, fimH, terC, fyuA, irp2, traT, iucC*
K.p21	ColKP3, IncFIB(K), ColRNAI, Col440I, IncHI1B, IncFIB(Mar)	21	***blaOXA-232,****blaSHV-28, blaSHV-106, catB3, blaOXA-1, aac(6')-Ib-cr, msrE,* ***armA****,sul1, dfrA12, aadA2, qacE, mphE, dfrA1, OqxB, OqxA, blaTEM-1A, tet(D), fosA, dfrA14, blaCTX-M-15*	*mrkA:ABW83989, iutA, fimH, terC, fyuA, irp2, traT, iucC*
K.p22	IncHI1B, IncFII(pHN7A8), ColRNAI, IncR, IncFIB(Mar),	36	***blaNDM-1,****blaKPC-2, OqxB, OqxA, msrE, aph(3')-VI,* ***armA****, qnrS1,sul1, sul2,mphE, mph(A), dfrA5, qacE,*	*mrkA:ABW83989, traJ, fimH, iutA, irp2, fyuA, terC, iucC,ccI*

			blaSHV-12, fosA, blaCTX-M-65	
K.p23	IncR, Col440II, Col(BS512), ColpVC, IncFII(pKPX1)	14	***blaNDM-5,*** *blaTEM-1B, aph(3'')-Ib, aph(6)-Id, sul2, sul1, qacE, qnrB1,mph(A), aac(6')-Ib-Hangzhou, aac(6')-Ib-cr,* ***rmtF****, OqxB, OqxA, dfrA12, aadA2, blaSHV-67, blaSHV-11, ARR-2, fosA, dfrA14, blaCTX-M-15*	*mrkA:ABW83989,iutA, fimH, fyuA, irp2, nlpI*
K.p24	IncHI1B, IncFIB(pKPHS1), Col440I, IncFIB(Mar)	12	***blaNDM-5****, OqxB, OqxA, sul2,* ***armA,*** *aph(3')-Ia, msrE, mphE, mph(A), dfrA5, qacE, sul1, catA1, blaSHV-67, blaSHV-11, fosA, aph(3')-VI, qnrS1, blaCTX-M-15*	*terC, iutA, iucC, fyuA, irp2, mrkA:ABW83989 fimH, nlpI*
K.p25	IncHI1B, IncFII(pHN7A8), ColRNAI, IncI1, IncFIB(Mar), IncR	32	***blaNDM-1,*** *blaKPC-2, blaCTX-M-65,blaSHV-182, erm(B), tet(X), sul2, fosA, mph(A), dfrA5, qacE, sul1,aph(3')-VI, qnrS1, aadA12,* ***armA****, aph(3')-Ia, mphE, msrE.*	*traJ, iucC, terC, ccI, fyuA, irp2, fimH, mrkA:ABW83989 iutA*
K.p26	IncHI1B, IncFII(pHN7A8), ColRNAI, ColKP3, IncR, IncFIB(Mar), Col440I	35	***blaNDM-1,*** *blaKPC-2, OqxB, OqxA, qnrS1, aph(3')-VI, sul2, blaCTX-M-65, fosA3, fosA, mph(A), dfrA5, qacE, sul1, blaSHV-182, mphE, msrE,* ***armA****, aph(3')-Ia*	*mrkA:ABW83989 fimH, fyuA, irp2, iutA, terC,traJ, ccI, iucC*
K.p27	ColKP3, IncFIB(K), IncHI1B, Col440I, IncFIB(Mar), ColRNAI	20	***blaOXA-232,*** *blaSHV-28, blaSHV-106, catB3, blaOXA-1, aac(6')-Ib-cr, msrE,* ***armA****,sul1, dfrA12, aadA2, qacE, mphE, dfrA1, OqxB, OqxA, blaTEM-1A, tet(D), fosA, dfrA14, blaCTX-M-15*	*mrkA:ABW83989 iutA, fimH, terC, fyuA, irp2, traT, iucC*
K.p28	IncFII(pHN7A8), ColRNAI, IncR, IncFIB(Mar), IncI1	36	***blaNDM-1,blaKPC-2,*** *OqxB, OqxA, erm(B), tet(X), qnrS1, aph(3')-VI,* ***armA****, aph(3')-Ia, mphE, msrE, fosA, mph(A), dfrA5, qacE, sul1, blaCTX-M-65, sul2, aadA12, blaSHV-12*	*mrkA:ABW83989 fimH, fyuA, irp2, iutA, terC,traJ, ccI, iucC*
K.p29	IncFII(pHN7A8), IncR, ColRNAI, IncHI1B, IncFIB(Mar), Col440I	25	***blaNDM-1, blaKPC-2,*** *OqxB, OqxA, blaSHV-182, sul2, fosA, fosA3,blaCTX-M-65, mph(A), aph(3')-VI, qnrS1, dfrA5, qacE, sul1, mphE, msrE, aph(3')-Ia,* ***armA,***	*mrkA:ABW83989 fimH, fyuA, irp2, iutA, terC,traJ, ccI, iucC*

K.p30	IncHI1B, IncFII(pHN7A8), ColRNAI, IncFIB(Mar), IncR, ColRNAI	34	***blaNDM-1, blaKPC-2,*** *OqxB, OqxA, blaSHV-182, sul2, fosA, fosA3,blaCTX-M-65, mph(A), aph(3')-VI, qnrS1, dfrA5, qacE, sul1, mphE, msrE, aph(3')-Ia,* ***armA,***	*mrkA:ABW83989 fimH, traJ, fyuA, irp2, terC, iutA, ccI, iucC*
K.p31	ColKP3, IncHI1B, Col440I, ColRNAI, IncFIB(K), IncFIB(Mar)	19	***blaOXA-232,*** *blaSHV-28, blaSHV-106, catB3, blaOXA-1, aac(6')-Ib-cr, msrE,* ***armA,*** *sul1, dfrA12, aadA2, qacE, mphE, dfrA1, OqxB, OqxA, blaTEM-1A, tet(D), fosA, dfrA14, blaCTX-M-15*	*mrkA:ABW83989 iutA, fyuA, irp2, iutA, terC, traT, fimH,iucC*
K.p32	ColKP3, Col440I, IncFIB(K), IncFIB(Mar), ColRNAI, IncHI1B	23	***blaOXA-232,*** *blaSHV-28, blaSHV-106, catB3, blaOXA-1, aac(6')-Ib-cr, msrE,* ***armA,*** *sul1, dfrA12, aadA2, qacE, mphE, dfrA1, OqxB, OqxA, blaTEM-1A, tet(D), fosA, dfrA14, blaCTX-M-16*	*mrkA:ABW83989 iutA, fyuA, irp2, iutA, terC, traT, fimH,iucC*
K.p33	ColKP3, ColRNAI, IncFIB(K), Col440I	14	***blaOXA-232,*** *blaSHV-28, blaSHV-106, fosA, OqxB, OqxA, blaCTX-M-15, dfrA1, aac(6')-Ib-cr*	*mrkA:ABW83989 iutA, fyuA, irp2, iutA, traT, fimH*
K.p35	ColKP3, IncFIB(K), IncHI1B, ColRNAI, Col440I, IncFIB(Mar)	21	***blaOXA-232,*** *blaSHV-28, blaSHV-106, catB3, blaOXA-1, aac(6')-Ib-cr, msrE,* ***armA***,*sul1, dfrA12, aadA2, qacE, mphE, dfrA1, OqxB, OqxA, blaTEM-1A, tet(D), fosA, dfrA14, blaCTX-M-16*	*mrkA:ABW83989 iutA, fyuA, irp2, iutA, terC, traT, fimH,iucC*
K.p36	IncFIB(pQil), IncHI1B, Col440I, IncFIB(Mar), IncR, IncFIB(pKPHS1)	16	***blaNDM-5,*** *blaTEM-1A, blaOXA-9, aadA1, aac(6')-Ib ,aac(6')-Ib-cr, OqxB, OqxA, catA1, msrE, aph(3')-VI,* ***armA,*** *qnrS1, mphE, mph(A), dfrA5, sul1, qacE, blaSHV-67, blaSHV-11, sul2, aph(3')-Ia, fosA, blaCTX-M-15*	*fimH, nlpI, mrkA:ABW83989 terC, irp2, fyuA, iutA, iucC*
K.p37	ColKP3, IncFIB(K), IncHI1B, ColRNAI, Col440I, IncFIB(Mar)	22	***blaOXA-232,*** *blaSHV-28, blaSHV-106, catB3, blaOXA-1, aac(6')-Ib-cr, , msrE,* ***armA***,*sul1, dfrA12, aadA2, qacE, mphE, dfrA1, OqxB, OqxA, blaTEM-1A, tet(D), fosA, dfrA14, blaCTX-M-16*	*mrkA:ABW83989 iutA, fyuA, irp2, terC, traT, fimH,iucC*

K.p38	IncFIB(Mar), IncFIB(pKPHS1), Col440I, IncFIB(pQil), IncR, IncHI1B	12	***blaNDM-5***, *blaTEM-1C, blaOXA-9, aadA1, aac(6')-Ib ,aac(6')-Ib-cr, OqxB, OqxA, catA1, msrE, aph(3')-VI,* ***armA****, qnrS1, mphE, mph(A), dfrA5, sul1, qacE, blaSHV-67, blaSHV-11, sul2, aph(3')-Ia, fosA, blaCTX-M-15*	*mrkA:ABW83989 iutA, fimH, fyuA, irp2, nlpI, iucC, terC*
K.p39	ColKP3, IncFIB(K), IncFIB(Mar), Col440I, IncHI1B, ColRNAI	19	***blaOXA-232,*** *blaSHV-28, blaSHV-106, catB3, blaOXA-1, aac(6')-Ib-cr,* ***armA,****sul1, dfrA12, aadA2, qacE, dfrA1, OqxB, OqxA, blaTEM-1A, tet(D), fosA, dfrA14, blaCTX-M-15*	*mrkA:ABW83989 iutA, fyuA, irp2, terC, traT, fimH,iucC*
K.p40	Col440I, IncHI1B, IncFIB(pKPHS1),IncFIB (Mar)	11	***blaNDM-5,*** *blaTEM-1B, blaOXA-9, aadA1, aac(6')-Ib, blaTEM-1C, aac(6')-Ib-cr, OqxB, OqxA, catA1, msrE, aph(3')-VI,* ***armA,*** *qnrS1, mphE, mph(A), dfrA5, sul1, qacE, blaSHV-67, blaSHV-11, sul2, aph(3')-Ia, fosA, blaCTX-M-15*	*mrkA:ABW83989 iutA, fyuA, irp2, terC, fimH,iucC*
