# Supplementary material for: The Evolving Clonal, Plasmid-Mediated Resistome, Virulome and Therapeutic Landscape of Carbapenem-Resistant Klebsiella pneumoniae in Oman
Source: Antibiotics (Basel). 2026 Aug 31;15(9):844. doi: 10.3390/antibiotics15090844 (PMC13603273; doi:10.3390/antibiotics15090844)
Supplement: Supplementary file 1 [file antibiotics-15-00844-s001.zip › Supplementary Table S3 CZA_AZT_Synergy_Table.pdf]

Supplementary Table S3 Synergistic Activity Between CZA and AZT

Carbapenemases	Strain ID	β-lactam family	Sample type	XDR/PDR	MLST	MIC		Cross Method		Fixed Ratio Method		MIC:MIC Method	
						CZA alone (S ≤8/4)	AZT alone (S ≤4)	FICI	Interpretation	FICI	Interpretation	ΣFICI	Interpretation
NDM-5	K.P 15	NDM-5	MSU	PDR	ST-147	256	48	0.16	Synergy	0.012	Synergy	0.005	Synergy
NDM-5	K.P 23	NDM-5	Abdominal wound	XDR	ST-147	256	24	0.07	Synergy	0.006	Synergy	0.002	Synergy
NDM-5	K.P 24	NDM-5	Wound	PDR	ST-147	256	256	0.16	Synergy	0.002	Synergy	0.001	Synergy
NDM-5	K.P 36	NDM-5	Swab	XDR	ST-147	256	256	0.15	Synergy	0.002	Synergy	0.002	Synergy
NDM-5	K.P 38	NDM-5	MSU	PDR	ST-147	256	256	0.17	Synergy	0.003	Synergy	0.003	Synergy
NDM-5	K.P 40	NDM-5	MSU	XDR	ST-147	256	256	0.16	Synergy	0.002	Synergy	0.002	Synergy
NDM-1+KPC-2	K.P 22	NDM-1, KPC-2	Peritoneal fluid	XDR	ST-11	64	256	0.54	Synergy	0.004	Synergy	0.234	Synergy
NDM-1+KPC-2	K.P 25	NDM-1, KPC-2	Tracheal Aspirate	XDR	ST-11	256	256	0.12	Synergy	0.002	Synergy	0.016	Synergy
NDM-1+KPC-2	K.P 26	NDM-1, KPC-2	Blood	XDR	ST-11	256	256	0.11	Synergy	0.008	Synergy	0.031	Synergy
NDM-1+KPC-2	K.P 28	NDM-1, KPC-2	Tracheal Aspirate	XDR	ST-11	256	256	0.17	Synergy	0.004	Synergy	0.016	Synergy
NDM-1+KPC-2	K.P 29	NDM-1, KPC-2	Tracheal Aspirate	PDR	ST-11	256	256	0.19	Synergy	0.016	Synergy	0.023	Synergy
NDM-1+KPC-2	K.P 30	NDM-1, KPC-2	Blood	PDR	ST-11	256	256	0.17	Synergy	0.012	Synergy	0.016	Synergy
KPC-2	K.P 5	KPC-2	Tracheal	XDR	ST-11	2	256	0.11	Synergy	0.378	Synergy	0.032	Synergy
